# A distributed cell division counter reveals growth dynamics in the gut microbiota

**DOI:** 10.1038/ncomms10039

**Published:** 2015-11-30

**Authors:** Cameron Myhrvold, Jonathan W. Kotula, Wade M. Hicks, Nicholas J. Conway, Pamela A. Silver

**Affiliations:** 1 Department of Systems Biology, Harvard Medical School, Boston, Massachusetts 02115, USA; 2 Wyss Institute for Biologically Inspired Engineering, Harvard University, Boston, Massachusetts 02115, USA

## Abstract

Microbial population growth is typically measured when cells can be directly observed, or when death is rare. However, neither of these conditions hold for the mammalian gut microbiota, and, therefore, standard approaches cannot accurately measure the growth dynamics of this community. Here we introduce a new method (distributed cell division counting, DCDC) that uses the accurate segregation at cell division of genetically encoded fluorescent particles to measure microbial growth rates. Using DCDC, we can measure the growth rate of *Escherichia coli* for >10 consecutive generations. We demonstrate experimentally and theoretically that DCDC is robust to error across a wide range of temperatures and conditions, including in the mammalian gut. Furthermore, our experimental observations inform a mathematical model of the population dynamics of the gut microbiota. DCDC can enable the study of microbial growth during infection, gut dysbiosis, antibiotic therapy or other situations relevant to human health.

## Abstract (web summary)

Research on the gut microbiota would benefit from improved methods to study microbial population growth. Here, Myhrvold *et al*. present a ‘mark and recapture' method that uses genetically encoded fluorescent particles to measure the growth rates of gut microbes in live animals.

## Graphical abstract

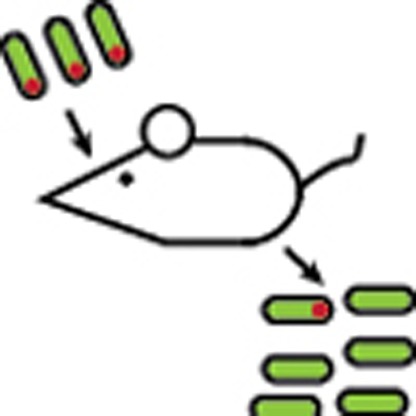

Animals rely on their associated microbial communities to aid with digestion, immunity, and other aspects of physiology^1^,^2^,^3^ and disease. To understand the structure and dynamics of the microbiota, researchers use metagenomics, metatranscriptomics and metabolic profiling^4^,^5^,^6^ to measure the response of the gut microbiota to changes in diet, health or environment^7^,^8^,^9^,^10^. These methods provide a birds-eye correlative view of the microbiota, but cannot be used to directly measure the growth rates of individual taxa. In particular, 16S recombinant DNA sequencing can only detect relative changes in frequency within a mixed gut population—a convolution of growth, death and competition with other microbes in the community. Recent work has indicated that the structure of the gut microbiota can change in <24 h in response to changes in diet or antibiotic therapy, but the mechanisms driving such changes remain largely mysterious^8^ because the *in vivo* growth characteristics of distinct gut microbes cannot be directly measured. To better understand the growth dynamics of the gut microbiota, it is paramount to deconvolve the effects of growth, death and competition, and thus new methods are needed to directly measure these quantities in the mammalian gut.

Mark and recapture, in which organisms are first marked and then sampled later, is used by ecologists to measure the growth rate of wild populations^11^. The fraction of individuals containing the mark on resampling allows for the change in population size to be estimated. Mark and recapture has been applied to study many animal populations, but methods for phenotypically marking bacteria (for example, superinfecting bacteriophage or temperature-sensitive plasmids) are limited by indirect measurement, stability and host range^12^,^13^,^14^,^15^. Such methods are capable of providing qualitative estimates of growth rate, but cannot be used to determine the number of elapsed generations precisely. In addition to phenotypic marking, sequence tagging methods such as wild-type isogenic tagged strains and sequence tag-based analysis of microbial populations have been recently developed^16^,^17^. These methods indirectly measure the growth rate of a population, but are very useful for elucidating population dynamics (such as population bottlenecks) during pathogen infection.

In addition to marker-based methods, markerless methods such as fluorescent hybridization to ribosomal RNA, and the peak-to-trough ratio based on next-generation sequencing have been used to study microbial populations where marking is not feasible^18^,^19^. Of these, the peak-to-trough ratio is especially exciting because it allows for the growth rates of many native microbiotal strains to be measured simultaneously from a metagenomics sample^19^. Furthermore, when genetic modification is possible, it often facilitates downstream measurement. For example, fluorescence dilution or the TIMER protein can be used to measure cell-to-cell heterogeneity in growth rate, without the expense of next-generation sequencing^20^,^21^. One limitation of fluorescence dilution and TIMER-based methods is that they work best in aerobic tissues due the oxygen-dependent nature of fluorescent protein maturation. As such, these methods have been primarily used to study *Salmonella* infection, which occurs in aerobic tissues^20^,^21^. For a detailed comparison of various methods for measuring *in vivo* population dynamics, see Supplementary Table 1.

Here we use a synthetic mark and recapture^11^ strategy at the microbial scale that enables us to count bacterial cell divisions. In contrast to synthetic stimulus counters, which move between predefined states in response to a series of chemical stimuli^22^, our method relies on inert particles that accurately segregate when cells divide, leading to an exponential decrease in the fraction of cells containing a particle over time. By measuring the distribution of particles in a population of cells, one can determine the number of elapsed generations. Thus, we call our method distributed cell division counting (DCDC).

## Results

### Implementing DCDC in *Escherichia coli*

**Figure 1:**
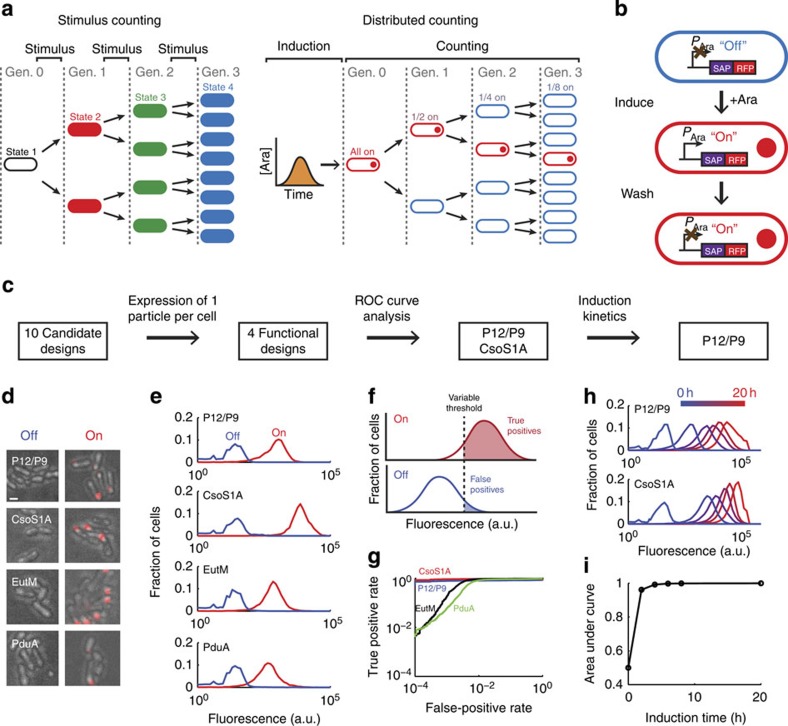
Implementing DCDC in *E. coli* cells. Stimulus counters switch between discrete states in response to specific triggers^22^,^35^, whereas distributed counters are induced and then an observable element (for example, a bright particle) autonomously segregates as cells divide (**a**). The number of elapsed generations is encoded as the ratio of the number of particles to the number of cells. DCDC was implemented using a self-assembling protein (SAP) fused to a red fluorescent protein (RFP) under control of an arabinose-inducible promoter (P_Ara_), producing monomers that self-assemble into a bright, fluorescent particle (**b**). After the cells are washed, particle production ceases but existing particles remain. We started with 10 designs for DCDC, and determined the best design as outlined in the schematic (**c**). To verify expression, cells were imaged after 3 h of induction with 1 mM arabinose using confocal microscopy (**d**, scale bar, 1 μm) and analysed using flow cytometry (**e**). The flow cytometry data from (**e**) were analysed using a range of thresholds between bright and dark cells (**f**) to create a receiver-operating characteristic curve^36^, which plots the true positive rate versus the false-positive rate for varying thresholds (**g**). The two best-performing designs were analysed by flow cytometry after an induction time course (**h**). To determine the optimal induction time, we calculated the area under the receiver operating characteristic curve for the P12/P9 design (**i**).

To implement DCDC (Fig. 1), we placed a series of self-assembling proteins (SAPs, which include bacteriophage shell proteins and bacterial microcompartment proteins) fused to a red fluorescent protein (mRFP1, hereby referred to as RFP) under control of the arabinose-inducible promoter in *E. coli* (Fig. 1b). After induction, ‘on' cells express a fluorescent fusion protein that self-assembles to form a single bright particle per cell; the inducer is then eliminated to prevent further particle production (Fig. 1b). We thus overcome the limitations of other microbial marking strategies by using inert, highly stable particles that do not confer any cellular growth burden and whose expression is tightly controlled by a single induction event. Chemical labelling methods, such as the dye Carboxyfluorescein succinimidyl ester, rely on analogue fluorescence measurements and are limited by the dynamic range of detection^23^,^24^,^25^ and heterogeneity in cell size. For these reasons, Carboxyfluorescein succinimidyl ester has been primarily used to study the proliferation of immune cells, which are large and homogenous in size. Unlike chemical labelling methods, DCDC is limited only by the number of cells counted and the false-positive rate (see below).

Individual, bright particles with distinct segregation characteristics can be expressed in *E. coli* (Fig. 1c). We designed 10 variants of DCDC using an arabinose-inducible promoter to express a variety of proteins known to self-assemble in bacterial cells, including bacteriophage and bacterial microcompartment (BMC) proteins^26^,^27^,^28^,^29^; 6 of these variants produced bright particles (Fig. 1d, Supplementary Fig. 1). Of these, four variants (P12–P9, CsoS1A, EutM, PduA) had one bright particle in the majority (60–80%) of cells after 3 h of induction (Fig. 1d). The other two variants (CbbL and T4.gp23) had multiple particles per cell or heterogeneous particle sizes (Supplementary Fig. 1). ‘On' cells were between 20- and 200-fold more fluorescent than ‘off' cells when measured by flow cytometry (Fig. 1e).

DCDC has a very low false-positive rate. To measure the performance of our DCDC designs, we calculated receiver operating characteristic curves from the flow cytometry data by systematically varying the threshold between ‘on' and ‘off' cells (Fig. 1f). We observed that two designs (P12/P9 and CsoS1A) had more than 1,000 true positives per false positive, which was much higher than the other two designs (EutM and PduA), which had about 100 true positives per false positive (Fig. 1g). To further winnow the number of designs, we compared the induction kinetics of the P12/P9 and CsoS1A designs. The fluorescence distribution increased monotonically for both designs, the P12/P9 design has slower kinetics than the CsoS1A design, which should result in fewer false positives for a given amount of spurious production (Fig. 1h). To determine the optimal induction duration, we calculated the area under the receiver operating characteristic curve. We used the induction time course data for the P12/P9 design from panel h to perform our analysis (Fig. 1i). This analysis indicated that we can classify ‘on' and ‘off' cells with an area under the curve >0.99 after 4 h of induction.

### Testing DCDC *in vitro*

**Figure 2:**
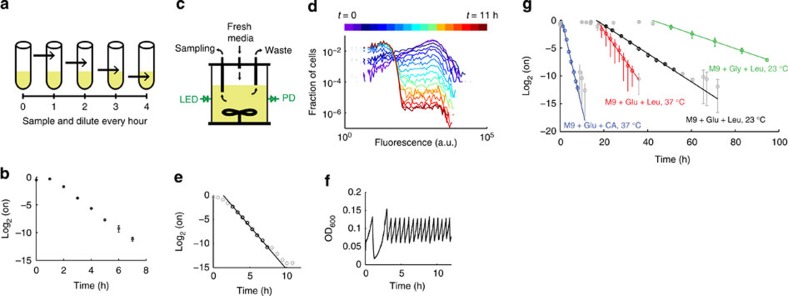
Testing DCDC *in vitro*. We maintained cells in exponential growth by diluting and sampling every hour (**a**). We plot the log_2_ of the fraction of ‘On' cells over time under standard culturing conditions that maintain exponential growth (**b**). Schematic of the custom turbidostat used for testing (**c**). Cells were grown in sealed glass bottles, mixed with a stir bar while fresh media inflow was controlled by an electromechanical valve. A constant volume was maintained using a gravity-fed waste line. Optical density was measured using a LED and photodiode, which fed back to control the fresh media valve. Samples were acquired from the turbidostats using a syringe. We calibrated individual turbidostats using serial dilutions of *E. coli* cultures with known optical densities (Supplementary Fig. 2b). Logarithmically plotted raw flow cytometry data (**d**), the log_2_ of the fraction of ‘On' cells over time (**e**) and optical density (**f**) are shown over 14 consecutive generations of exponential growth in minimal media supplemented with 0.4% (w/v) glucose, 0.5% (w/v) casamino acids and 100 mM potassium nitrate. In panel (**d**), the data are plotted on a log-log scale. A line was fit to the black circles to determine the doubling time. We show the log_2_ of the fraction of ‘On' cells across a wide range of growth rates and temperatures with *n*=3 replicates for each temperature and condition (**g**). Leucine was supplemented because the parent strain (DP10) is a leucine auxotroph. Error bars indicate one standard deviation. Lines were fit to the coloured circles to determine doubling times. CA, casamino acids; Glu, glucose; Gly, glycerol; Leu, leucine.

DCDC can track at least 10 consecutive cell divisions during exponential growth. We tested DCDC by inducing cells for 4 h with arabinose, washing them 3 × with phosphate-buffered saline (PBS), then diluting 1:1,000 in fresh medium. To maintain exponential growth, cells were diluted every 2 h into flasks containing fresh Lysogeny Broth (LB) medium (Fig. 2a). As expected, we observed an exponential decreases in the fraction of ‘on' cells over time after a short lag phase (Fig. 2b). We further tested DCDC using a custom-built turbidostat (Fig. 2c, Supplementary Fig. 2a) to automate growth curve collection and maintain cells in exponential growth for a defined number of generations. After 4 h of induction in arabinose-containing rich medium, ∼70% of cells were in the ‘on' state. The cells were then washed and diluted into defined medium to terminate particle production, thereby allowing us to track cellular divisions. We measured the fluorescence of cells at discrete time points using flow cytometry (Fig. 2d, data are plotted on a log–log scale) and calculated the fraction of ‘on' cells over time (Fig. 2e). The bacterial populations initially take one or two generations to adapt to the new growth conditions after which cultures reach maximal exponential growth rates. By fitting a line to the log_2_ of the fraction of ‘on' cells, we calculate a doubling time of 33.8 min, which is only about 3% longer than the doubling time based on optical density (32.9 min, Fig. 2f). This difference corresponds to less than one half of one doubling over the course of the experiment.

DCDC is robust to large changes in growth rate. We varied the carbon source and temperature and measured the change in bright fraction over time in turbidostats (Fig. 2g). We fit an exponential decay (lines) to the coloured points during which cells were growing exponentially. Across this wide range of temperatures and conditions, the fraction of ‘on' cells decreased exponentially, as expected. At slower growth rates, the doubling time calculated from slope of the log_2_ of the fraction of ‘on' cells was longer than the doubling rate as measured by optical density, but this can be accounted for using a quadratic calibration curve (Supplementary Fig. 2d). Thus, DCDC can measure generation times across an order of magnitude (0.5–5 h), which corresponds to a 512-fold difference in population size per generation (2^10^/2^9^=512).

### Detailed analysis of the performance of DCDC

**Figure 3:**
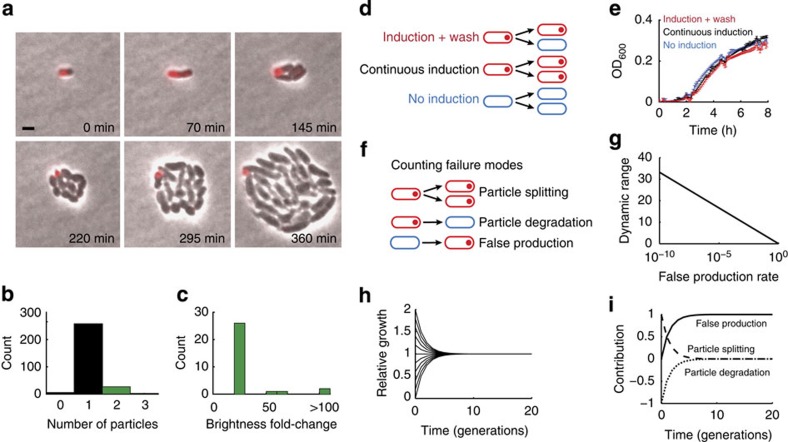
Quantitative analysis of distributing cell division counting. To analyse the performance of DCDC, we directly observed microcolony formation under an agar pad using time-lapse microscopy. Overlays of phase (grey) and RFP (red) are shown during microcolony formation (**a**). Scale bar, 2 μm. In each frame of *n*=4 movies, we measured the number of bright particles in each frame (**b**). In frames with multiple bright particles, we calculated the fold-change between the brightest and second-brightest particle in a frame (**c**). To measure the growth burden of DCDC, we constantly induced, induced and washed, or did not induce cells (**d**) and measured growth curves after each of these treatments (**e**). Error bars indicate one standard deviation. We created a mathematical model to understand the effects of particle splitting, degradation and false production (**f**) on the performance of the counter. Using our model, we calculated the dynamic range in generations as a function of the false production rate (**g**), and the effect of growth disparities between bright and dark cells over time (**h**). We also determined the relative contributions of particle splitting, degradation and false production to counting error over time (**i**).

DCDC particles segregate faithfully. We observed exponentially growing cells using an agar pad on top of a coverslip using time-lapse fluorescence microscopy. Over time, individual, labelled cells formed microcolonies in which only one cell was labelled with a counting particle (Fig. 3a; Supplementary Movie 1). We determined the extent of particle splitting and false production by measuring the number of fluorescent particles in each frame of the movie. In 90% of frames, only one bright particle was observed (Fig. 3b). In the remaining 10% of frames, one ‘main' bright particle and one or two dim particles were observed. We calculated the ratio of brightness between the brightest and second-brightest particles in all of the frames with at least two particles, and found that the fold change between the main particle and the next brightest particle was always >20 (Fig. 3c). As a result, we could differentiate between bright, positive cells and false positives. In most cases, the dim particles originated from cells that did not contain a particle, suggesting in rare cases that these extra particles are being produced *de novo* rather than splitting from existing particles. Together, this analysis suggests that DCDC is robust to segregation errors and false particle production.

DCDC does not incur a fitness cost on cells. We grew cells under three different conditions: no induction, continuous induction and induction followed by a wash (Fig. 3d) to determine whether particle expression affected growth rate. After growth in the presence or absence of inducers, we diluted cells 1:1,000 in fresh medium and measured their subsequent growth by monitoring the optical density 600 (OD_600_) over time (Fig. 3e). We observed similar growth curves under all three conditions, indicating that particle expression does not affect the growth rate of cells. Thus, we conclude that particle production does not introduce a growth burden on cells.

DCDC is sensitive to false production but not particle splitting, degradation or differential growth rates. We created a mathematical model in which particle splitting, degradation and false production occur in exponentially growing cells (Fig. 3f and Supplementary Fig. 3). We used our model to determine the maximum dynamic range in generations and found that the dynamic range was inversely proportional to the log_2_ of the false production rate (Fig. 3g). We also examined the effect of growth-rate difference between ‘on' and ‘off' cells, and determined that such growth rate differences have only a transient effect on the average growth rate of the population because the fraction of ‘on' cells decreases exponentially over time (Fig. 3h). Thus, the performance of DCDC is not sensitive to differences in growth rate between ‘on' and ‘off' cells, and as established in Fig. 3e such differences do not exist in practice. Finally, we examined the relative contributions of particle splitting, particle degradation and false particle production to the counting error. We found that particle splitting and degradation are important for the first few generations, but in the long run, false production dominates as a potential error source (Fig. 3i), which could lead to underestimates of the actual growth rate (Supplementary Fig. 2d). However, our modelling indicates that the maximum false production rate 
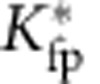
 is equal to the steady-state on/off ratio (see Methods section for details), which based on our turbidostat experiments (Fig. 2e,g) is typically less than 1 in 1,000. Therefore, we do not expect false production to have a large effect on counting accuracy when the on/off ratio is greater than 
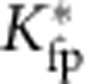
.

### Using DCDC to measure the growth of gut microbes

**Figure 4:**
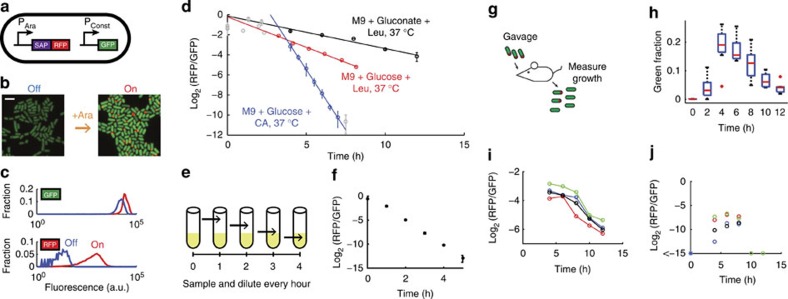
Engineering gut microbes to count cell divisions. GFP and RFP expression from the engineered gut *E. coli* strain PAS418 (**a**) was verified using confocal microscopy (**b**) and flow cytometry (**c**) after overnight growth with 133 mM arabinose. Scale bar in **b**, 2 μm. DCDC was tested in PAS418 using growth in a turbidostat in minimal media supplemented with carbon sources and amino acids as indicated (**d**). Error bars indicate one s.d. based on two or three replicate turbidostats (there was only one replicate for the M9+Glu+Leu condition). We also measured the growth of PAS418 under more standard culturing conditions by periodically diluting cultures grown in flasks in LB medium (**e**–**f**). PAS418 cells were introduced into mice by oral gavage and the growth was monitored using DCDC by collecting faeces every 2 h (**g**–**j**). We first measured the average transit time by monitoring the presence of GFP^+^ bacteria in the faeces of *n*=8 mice, shown in a box and whisker plot (**h**). The edges of each box are the 25th and 75th percentiles, and the middle is the median. The whiskers extend to the most extreme data points that are not considered outliers. Outliers are indicated using red crosses. We then measured the growth of induced (**i**) and uninduced (**j**) PAS418 cells in the mouse gut by plotting the log_2_ of the red/green fraction from *n*=4 mice. In **i**, data points are joined by lines to aid the eye.

DCDC can be implemented in gut microbes. We modified a naturally occurring *E. coli* strain isolated from the mouse gut^30^ to contain a DCDC plasmid and a chromosomally integrated constitutively active superfolder *green fluorescent protein* (sf *GFP*) gene, yielding the strain PAS418 (Fig. 4a). This enabled us to distinguish our engineered microbes from the native gut microbiota by flow cytometry or microscopy. PAS418 cells expressed RFP particles (Fig. 4b, Supplementary Fig. 4a) after induction with arabinose. Background particle expression was low (0.15 %) in the absence of induction, as measured by flow cytometry (Fig. 4c). The efficacy of DCDC in PAS418 was first determined *in vitro* by growing PAS418 in our custom turbidostat under a variety of growth conditions and measuring growth rates over eight generations (Fig. 4d, Supplementary Fig. 4b). As was observed with a lab *E. coli* strain (Fig. 2g), the fraction of ‘on' cells decreased exponentially over time across several different growth conditions. We also tested DCDC by periodically diluting cells grown in flasks in LB at 37 °C (Fig. 4e), and the device functioned as expected (Fig. 4f). Thus, DCDC is functional in PAS418, a naturally occurring mouse gut bacteria.

We used DCDC to directly quantify the dynamics of the microbiota during colonization in the gut. To determine the relative influences of growth, death and removal on microbiotal populations, we conducted a series of experiments in which PAS418 cells were introduced into mice by oral gavage and their growth rate was subsequently measured using DCDC by sampling the faeces every 2 h after gavage (Fig. 4g). We first determined the rate of removal by monitoring the fraction of bacteria in the faeces that were GFP^+^ every 2 h after gavage (Fig. 4h). Green (GFP^+^) bacteria were first observed 4 h after gavage, and after 6 h the fraction of green bacteria decreased. Thus, the median transit time was 6 h corresponding to a removal rate of 1/6 h^−1^.

Microbes can divide rapidly in the mammalian gut. We measured the growth rate of PAS418 in the mouse gastrointestinal tract by introducing induced PAS418 cells into *n*=4 mice by oral gavage (Fig. 4i, Supplementary Fig. 4c). We fit lines to the log_2_ of the red/green fraction, and found that the doubling time was 2.91±0.06 h. As a negative control, we introduced uninduced PAS418 cells into *n*=4 mice by oral gavage, and observed that fewer than 1% of bacteria were reinduced in the gastrointestinal tract based on our measurements of the log_2_ of the R/G fraction (Fig. 4j, Supplementary Fig. 4d). Data from a replicate experiment indicated a similar doubling time of 2.74 h (Supplementary Fig. 5).

### Mathematical modelling of growth dynamics in the gut

**Figure 5:**
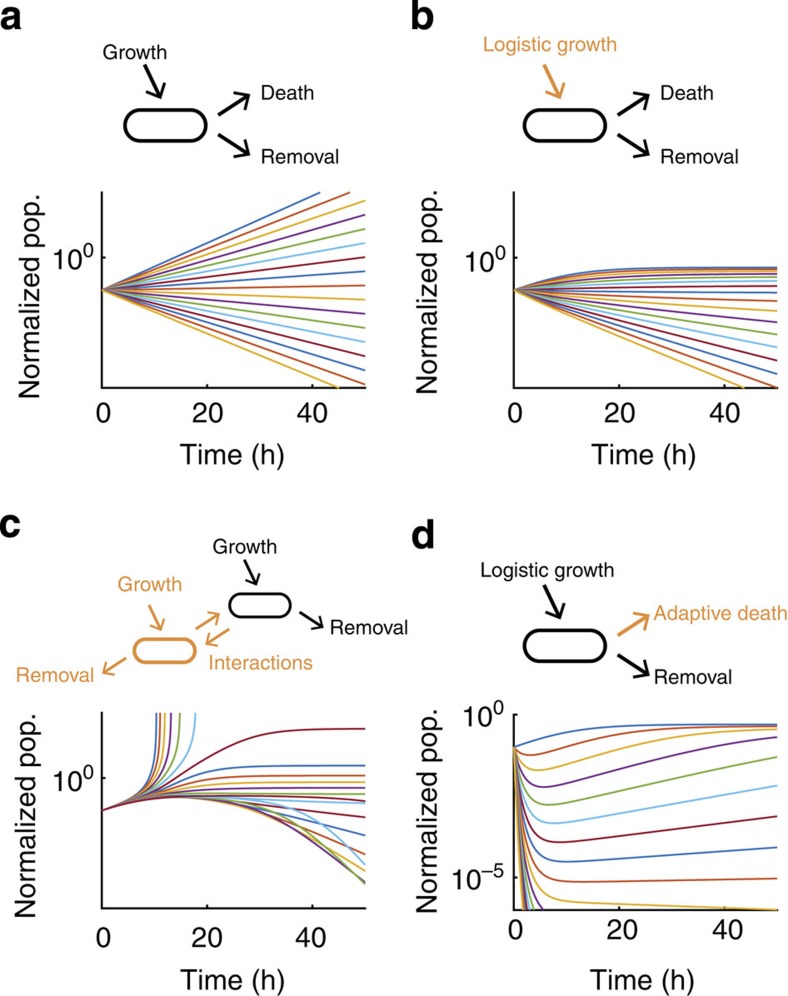
A simple model of the population dynamics of the gut microbiota. We first consider a model with constant growth and removal rates and variable death rates from 0 h^−1^ to 1/3 h^−1^ (**a**). We then consider a model with logistic growth, a constant removal rate and variable death rates from 0 h^−1^ to 1/3 h^−1^ (**b**). Finally, we consider a model with two separate species, each of which grows logistically and has a constant removal rate (**c**). In this model, death is mediated by positive or negative interactions between the species, which are systematically varied from −1 (strong, negative interactions) to 1 (strong, positive interactions). We also consider a model with logistic growth, constant removal and a death rate that exponentially decays to a constant rate (**d**). Here we vary the final death rate from 0 h^−1^ to 1/3 h^−1^.

Our experimental data capture the growth dynamics during the initial phase of colonization in the gut, but over the next few days, the steady-state population size of PAS133 (the gut isolate we engineered to enable DCDC) is much lower than the initial population size^30^. To investigate the forces that might shape the long-term population dynamics of PAS418 and related strains in the mammalian gut, we constructed a mathematical model. The simplest version of the model accounts for growth (at a rate of 1/3 h^−1^, based on our measurements), removal (at a rate of 1/6 h^−1^, based on our measurements) and death (an unknown parameter that we vary in the model) using differential equations (Fig. 5a). Such a model is inherently unstable (aside from a trivial stable solution where the population size is zero); the population will either grow or decay exponentially over time, unless the growth, death and removal rates balance each other, which is a bifurcation point.

Our simplest model does not consider nutrient limitation in the gut, so we introduced a logistic growth term to account for this (Fig. 5b). Such a model does produce a steady-state population, but only when the population initially increases in size or remains constant, which is inconsistent with experimental observations that the steady-state population size of PAS133 is much lower than the initial population size^30^. Furthermore, we know that the bacteria do not initially start above their carrying capacity because they are able to grow rapidly during the first 12 h after gavage (Fig. 4i), and we introduce the bacteria at a density of 10^8^ ml^−1^, which is about 10 times lower than the estimated carrying capacity of 10^9^ c.f.u. ml^−1^, based on previous measurements of bacterial loads in mouse faeces^30^,^31^.

Competition between bacteria does not explain the observed population dynamics. We introduced a second bacterial species to our model that grows logistically and is removed at a constant rate. In this model, mutual interactions between the two species can be antagonistic or sympathetic, and we systematically vary the strength and sign of the interactions. For simplicity, we consider symmetric interactions, and death is mediated by interactions between the strains. We show the population dynamics for one of the species over time with varying interaction strengths and signs (Fig. 5c). Broadly, the results fall into three categories: both species grow exponentially, both species decline exponentially and a regime in which both species exist in the steady state. As in Fig. 5b, stability is only observed when the population does not initially decline over time. Thus, competition does not produce dynamics that are consistent with our observations.

Finally, we considered the effects of adaptation on the population dynamics of gut microbes. In this model, some bacteria alter their gene expression over time such that the steady-state death rate is lower than the initial death rate. This means that initially, the population decreases in size, but eventually stabilizes as the microbes adapt to their new environment (Fig. 5d). The extent of the initial drop in population size depends on the initial death rate, which we varied from 0 to 10 h^−1^. If the initial death rate is low, the initial population drop is small, and eventually the population recovers to its carrying capacity, whereas if the long-term death rate (which we varied from 0 to 1/3 h^−1^) is too high, the population crashes. In between these extremes, the model produces population dynamics that are qualitatively similar to experimental observations that several days after gavage, PAS133 stabilizes at a low population level and maintains that population level for a long time^30^. However, the model in Fig. 5d does not consider competition with other species, or other forces that could shape the exact size the population reaches in the steady state. A model with adaptation is able to qualitatively reproduce the observed population dynamics of the gut microbiota, but models without adaptation only do so with a narrow range of initial conditions. This suggests that the dynamics of death could be a key difference between the initial population that colonizes the gut and the population that resides there in the long term.

## Discussion

In summary, we have developed a new method, DCDC, which allows us to infer the population dynamics of microbes in a natural setting where birth and death are not directly observable. Mathematical modelling indicated that the ability to draw such inferences depends crucially on non-replication of the mark and from extremely low false-positive and false-negative measurement rates. Previously, Benjamin *et al*.^32^ attempted to use a temperature-sensitive episomal element as a non-replicating mark in *Salmonella*, but could not quantitatively measure growth rates because replication was not completely shut off at body temperature. Using our system, we found that a murine *E. coli* strain residing in the mouse gut divided twice as fast as the removal rate by defecation. This, combined with previous measurements of the small steady-state population size of the PAS133 strain^30^, implies that a significant number of PAS418 cells in the gut are lost to death. Furthermore, the long-term stability of PAS133 in the gut suggests that it may be able to adapt to the conditions in the gut, as indicated by our model (Fig. 5d). It is also possible that adaptation of the removal rate could lead to a steady-state population in the gut, but this is less biologically plausible. The removal rate is largely determined by the frequency and amount of defecation, which is a function of diet and gut physiology. It is also conceivable that nutrient limitation alone could lead to an initial decrease in population size followed by stability, but this only is feasible when the initial population size is near the carrying capacity. Since we introduced our bacteria at about 1/10 of the carrying capacity, the initial conditions for the nutrient limitation model are not satisfied. More generally, it is unlikely that a newly colonizing bacterial species will have an initial population size close to the carrying capacity, as this would imply that colonization has already taken place. That said, it is important to emphasize that our modelling is not exhaustive—other models could explain the observed population dynamics—but rather serves to illustrate a possible scenario that could explain our experimental observations.

Our method should be easily applied to other species in the microbiota so long as the species can be genetically modified to express a self-assembling protein fusion under control of an inducible promoter. Obligate anaerobic bacteria will be a particular challenge due to the use of oxygen-dependent fluorescent proteins, but this could be circumvented using a non-oxygen-dependent fluorophore such as the iLOV protein^33^. In addition, the dynamic range of DCDC is set by the number of generations, so should function in microbes with longer colonization times or slower growth rates. Through additional experimental and computational investigations using DCDC and other methods, it should be possible to further elucidate the complex population dynamics of the gut microbiota.

## Methods

### Strains, plasmids and bacterial culturing

For a list of strains and plasmids used in this study, see Supplementary Table 2. For plasmid maintenance, ampicillin was used at a final concentration of 100 μg ml^−1^, streptomycin was used at a final concentration of 300 μg ml^−1^. Unless indicated otherwise, cells were grown in LB media. Plasmids (see Supplementary Fig. 7 for a reference plasmid map) were constructed using PCR with Q5 or Phusion polymerase (New England Biolabs) and Gibson assembly (see Supplementary Table 3 for a list of primer sequences used). QIAprep spin or QuickLyse kits (Qiagen) were used for isolating DNA, and DNA Clean & Concentrator-5 kits (Zymo Research) were used for PCR purification. Plasmid inserts were confirmed via Sanger sequencing (Eton Bioscience).

### *In vitro* counting experiments

*E. coli* DP10 cells were grown overnight in LB medium supplemented with ampicillin and back-diluted 1:100. After 1.5–2 h of growth, cells were induced for 3–4 h with 1 mM arabinose, then washed once with 1 × PBS and concentrated 10-fold by centrifugation. 1 ml of cells was inoculated into 1 l of medium (described below) in a turbidostat bottle. In some experiments, we grew cells exponentially in LB medium in flasks (shaken at 250 r.p.m. at 37 °C), and diluted cultures 1:4 (for DP10) or 1:8 (for PAS418) into fresh, pre-warmed LB medium every hours to maintain exponential growth.

To monitor bacterial growth over many consecutive generations, we constructed a home-made turbidostat, which we named the ‘Evolvulator'. Each Evolvulator consisted of an Ethernet microcontroller fitted with a custom-designed daughterboard capable of connecting to and controlling various components (see Supplementary Tables 4 and 5 for parts lists). An light-emiting diode (LED) and photodiode were used to measure the OD of cells grown in a 1 l glass bottle. Since the 1 l bottle has a light path length of ∼10 cm, we chose a LED that emits light at 527 nm to minimize absorption of the light by the water. Cells were stirred using a magnetic stir bar inside the bottle and a magnet mounted on a computer fan; stirring speed was modulated using a potentiometer (POT0) to control voltage to the fan via pulse width modulation. The Evolvulator chassis was constructed from laser-cut 3 mm acrylic. Fresh medium was supplied by gravity flow from a 20 l carboy, governed by an electromechanical pinch valve. Custom server software written in Python and utilizing the Twisted event-driven network engine was developed to control a feedback loop, run on a Zotac ZBOX HD-AD02 with Ubuntu 11.10 installed. A web interface was constructed using Java and HTML, allowing the user to monitor OD in real time, as well as modify experimental parameters such as target OD, variance around target OD, and blank OD sensor reading. Photodiode readings and valve state information were saved to a SQL database stored locally on the control server. Samples were collected from the bottle using a syringe at regular intervals. All server code and design schematics can be accessed at the following GitHub repository: (see Supplementary Software 1, https://github.com/Wyss/evolvulator.git).

The LED on each Evolvulator device was calibrated via potentiometer adjustment (POT1) to a single photodiode to ensure that the intensity of emitted light was comparable between devices. After LED calibration, each device-specific photodiode was then calibrated against an in-house NanoDrop 2000c spectrophotometer (Thermo Scientific) to eliminate device-to-device differences on OD readings due to inherent manufacturing tolerances of the electrical components. Specifically, calculated Evolvulator ODs were plotted against NanoDrop ODs to generate a calibration curve (Supplementary Fig. 2b). Since the relationship of Evolvulator OD to NanoDrop OD was not linear, we fitted a polynomial trend line to the data that was forced through the origin. The resulting device-specific coefficients were then used to calculate NanoDrop equivalent ODs from raw sensor data collected during a given experiment. We wrote custom Python and Matlab scripts to extract and analyse photodiode sensor and valve activity data from the databases generated during an experiment. Generation times were calculated after every bioreactor dilution event. Briefly, % transmittance was calculated using equation (1). NanoDrop equivalent OD was then calculated using equation (2). The Ln(OD) was then plotted and the slope of this exponential growth curve was extracted using the polyfit Matlab function. Generation times were then calculated using equation (3).

Equations:


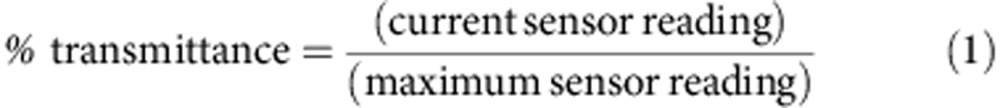



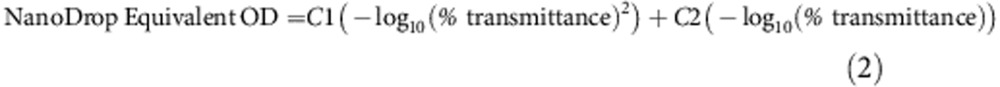


*C*1 and *C*2 in equation (2) represent the device-specific coefficients calculated from device calibrations.


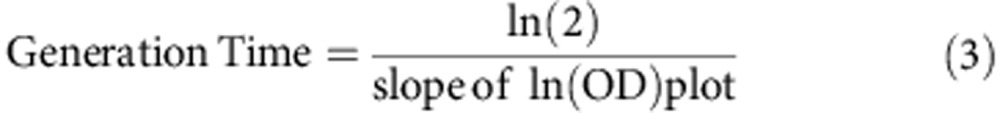


For the experiments shown in Fig. 2b,c, and Supplementary Fig. 2c, we used a minimal media containing 135.6 g of disodium phosphate, 60 g of monosodium phosphate, 10 g of sodium chloride, 20 g of ammonium chloride, 80 g of glucose (0.4% w/v), 100 g of casamino acids (0.5% w/v), 202.2 g of potassium nitrate (final concentration: 100 mM), per 20 l carboy. The media was supplemented with magnesium sulfate (final concentration: 1 mM), thiamine hydrochloride (final concentration: 1 μg ml^−1^), and calcium chloride (final concentration: 100 μM).

For the experiments shown in Fig. 2d and Supplementary Fig. 2d, we used M9 salts supplemented with potassium nitrate (final concentration: 100 mM), magnesium sulfate (final concentration: 1 mM), thiamine hydrochloride (final concentration: 1 μg ml^−1^) and calcium chloride (final concentration: 100 μM). We varied the carbon source, amino acid mixture and temperature. We used the following combinations: glucose (0.4% w/v) and casamino acids (0.5% w/v) at 37 °C, glucose (0.4% w/v) and leucine (final concentration: 1 mM) at 37 °C, glucose (0.4% w/v) and leucine (1 mM final concentration) at 23 °C, glycerol (0.4% w/v) and leucine (final concentration: 1 mM) at 23 °C.

For the experiments shown in Fig. 4d, we used M9 salts supplemented with glucose (0.4% w/v), casamino acids (0.5% w/v), potassium nitrate (final concentration: 100 mM), magnesium sulfate (final concentration: 1 mM), thiamine hydrochloride (final concentration: 1 μg ml^−1^), and calcium chloride (final concentration: 100 μM). Cells were grown at 37 °C.

For the experiments shown in Fig. 4d and Supplementary Fig. 4b, we used M9 salts supplemented with potassium nitrate (final concentration: 100 mM), magnesium sulfate (final concentration: 1 mM), thiamine hydrochloride (final concentration: 1 μg ml^−1^), and calcium chloride (final concentration: 100 μM). We varied the carbon source, amino acid mixture and temperature. We used the following combinations: glucose (0.4% w/v) and casamino acids (0.5% w/v) at 37 °C, glucose (0.4% w/v) and leucine (final concentration: 1 mM) at 37 °C, sodium gluconate (0.4% w/v) and leucine (final concentration: 1 mM) at 37 °C.

For the experiments shown in Supplementary Fig. 2d, we compared the doubling time based on optical density (τ_OD_, calculated as described above) with the doubling time based on the decrease in the fraction of ‘on' cells (τ_ON_, calculated based on the slope a linear fit to the log_2_ of the fraction of ‘on' cells, using the red data points). We observed an initial adaptation to new growth conditions (slower decrease in the ‘on' fraction) at the beginning of each time course. We also observed a slower decrease in the ‘on' fraction towards the end of each time course, due to either spontaneous particle production or false positives. If no particles are being produced after induction, we expect the two measures of doubling time to be identical, namely τ_OD_=τ_ON_. However, particle production (via leaky expression or particle splitting) will generally cause τ_ON_ to be larger than τ_OD_. We introduce a term *α* to account for this production, which is defined as the difference in growth rates as calculated by optical density and DCDC, such that *α*+1/τ_ON_=1/τ_OD_. In Supplementary Fig. 2d, we calculated *α*=1/24.

### *In vivo* counting experiments

*E. coli* PAS133 was transformed with pCAM10A, then the chromosomally integrated sfGFP construct was transferred via *P1vir* transduction from *E. coli* PAS143, a strain characterized by Brian Chin (Harvard Medical School; current affiliation, Sysmex Inostics; unpublished). This new strain was termed PAS418.

Female, 10-week-old, BALB/c mice were obtained from Charles River Laboratories, and allowed to acclimate for 1 week. Orally administered *E. coli* generally will not colonize the gut unless the endogenous bacteria are inhibited. Therefore, supplemented the drinking water of the mice with 5% sucrose and 0.5 mg ml^−1^ streptomycin to reduce the endogenous flora 1 day before oral administration of engineered *E. coli*^34^. Engineered *E. coli* were cultured overnight in M9 supplemented with 0.4% (w/v) glucose, 0.5% (w/v) casamino acids and ampicillin. We used 2% (w/v) arabinose for overnight induction. Approximately 10^7^ engineered *E. coli* cells from the overnight culture were washed in PBS and administered to each mouse via oral gavage. Faecal samples were collected every two hours by isolating mice in sterile plastic containers for ∼3 min until at least three faecal pellets were produced. Faecal pellets were suspended in 1 ml PBS, diluted 1:10 in PBS and centrifuged at 50*g* for 20 min. 600 μl of supernatant was removed and stored at 4 °C until flow cytometry analysis, which was performed within 24 h of sample collection. Throughout all experiments, mice were fed a grain-based chow without arabinose (ssniff EF R/M Control feed, ssniff-Spezialdiäten GmbH, Soest, Germany) *ad libitum.* Throughout our experiments, the animals did not present any signs of pain or stress. Our animal protocol was approved by the Harvard Medical Area Standing Committee on Animals, protocol 04966.

### Growth rate comparisons

*E. coli* DP10 and PAS418 cells were grown overnight in LB medium. PAS418 cells were induced overnight with 2% (w/v) arabinose, and DP10/pCAM10 cells were induced for 4 h with 1 mM arabinose. Cells were washed three times in 1 × PBS, and then diluted 1:1,000 in fresh LB medium. 150 μl of diluted cells were transferred to 96-well microplates (Corning) with 100 μl of mineral oil overlaid on top to prevent evaporation. The plate was incubated in a Perkin Elmer Victor 3 V 1420 multilabel plate reader at 37 °C and medium shaking was performed for 8 h, and the optical density at 600 nm was recorded every 5 min.

### Confocal microscopy

*E. coli* cells were imaged using a Nikon Ti motorized inverted microscope equipped with 100 × Plan Apo NA 1.4 objective lens, combined with a Yokagawa CSU-X1 spinning disk confocal with Spectral Applied Research Aurora Borealis modification. For mRFP1 imaging, a 100 mW 561 nm solid state laser with a quad pass dichroic mirror (Chroma) and a 620/60 emission filter (Chroma #858). For GFP imaging, a 488 nm solid state laser with a quad pass dichroic mirror (Chroma) and a 525/50 emission filter (Chroma #852) was used. Imaging was performed with a Hamamatsu ORCA-AG cooled charge-coupled device camera. Metamorph software was used for image acquisition. For *z*-stacks, seven optical sections with a spacing of 0.5 microns were acquired, and are displayed as maximum *z*-projections. Brightness and contrast were adjusted uniformly using ImageJ version 1.48. These images are shown in Figs 1 and 4, Supplementary Figs 1 and 4

### Time-lapse microscopy

Samples were placed in a MatTek dish, covered by a 2% agarose pad containing M9 medium supplemented with 0.4% (w/v) glucose and 0.5% (w/v) casamino acids. A low density of cells was used to allow for the formation of well-separated microcolonies. Time-lapse images were acquired using a Nikon TE-2000 microscope with a 100 × 1.4 numerical aperture phase objective with an ORCA-ER charge-coupled device camera (Hamamatsu Photonics, Hamamatsu, Japan). Illumination was provide from a Lumencor LED fluorescence illuminator. NIS-Elements AR version 4.20.00 was used to control the microscope and camera during acquisition. In general, we acquired frames every 5 min using no more than 50% of the maximum power on the Lumencor to minimize the effects of photobleaching. These images are shown in Fig. 3.

### Flow cytometry

*E. coli* cells were analysed using a BD LSRII flow cytometer with a High-Throughput Sampler. A 594 nm laser and 630/22 filter was used for mRFP1 detection. A 488 nm laser and 525/50 filter was used for GFP detection. Cells were gated by forward and side scatter to exclude doublets. Samples were run at ≤2,000 events per s to ensure accurate detection. When necessary, cells were concentrated by centrifugation at 5,000*g* for 10 min and the resulting pellet resuspended in a smaller volume. For the *in vitro* experiments shown in Fig. 2, tubes were used; the high-throughput sampler was used in other experiments. Control experiments were performed with alternating bright and dark wells to ensure minimal carryover between wells. Flow cytometry data were analysed using FlowJo v. 10.6, or custom MATLAB scripts (Mathworks, Natick, MA; Supplementary Software 2).

### Error rate modelling

We constructed a mathematical model to better understand the effects of particle degradation, splitting or false production on counting error. This model considers particles and cells separately. Suppose we have *p* particles in *n* cells. Then we can write down the following differential equations to describe the dynamics of particles and cells:


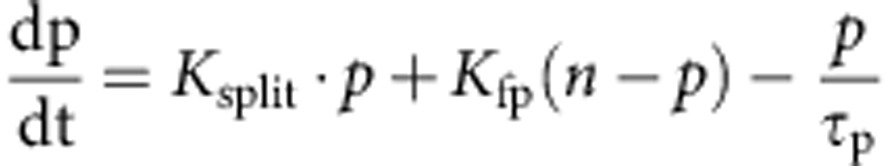



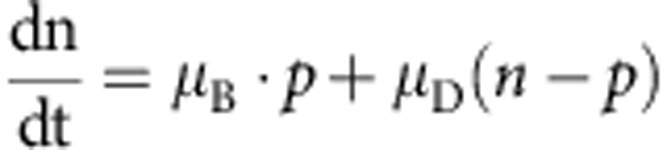


In the above equations, *K*_split_ is rate at which particles split, *K*_fp_ is the rate at which particles are produced in the absence of induction, *τ*_p_ is the inverse of the rate of particle decay, *μ*_B_ is the growth rate of bright cells, and *μ*_D_ is the growth rate of dark cells. For a reaction diagram, see Supplementary Fig. 3.

In the model, we assume that there can only be one particle per cell. Hence, there are *p* cells which contain particles, and *n*−*p* which do not contain particles. We denote these as ‘bright' and ‘dark' cells, respectively. Using the model, we can calculate (1) the dynamic range of DCDC, (2) the effect of unequal growth rates between bright and dark cells and (3) the relative contributions of particle splitting, degradation and false production over time.

In order for counting to function, we must have a separation of timescales between the particles and the cells. If cellular dynamics do not occur faster than particle dynamics, then the ratio of particles to cells will reflect production, degradation or splitting of the particles rather than growth and division of the population of cells being measured. In other words, the cells must be growing much faster than the net production (or decay) of particles. Based on our time-lapse microscopy and turbidostat experiments, we have observed that particles are stable for multiple days, and that particle production is much slower than cell division. Thus, we can safely assume that as 
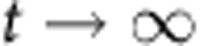
, 
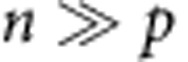
.

(1) To determine the dynamic range, we calculate the steady-state ratio pf *p*/*n* at time goes to infinity. To start, we normalize time relative to the growth rate of dark cells


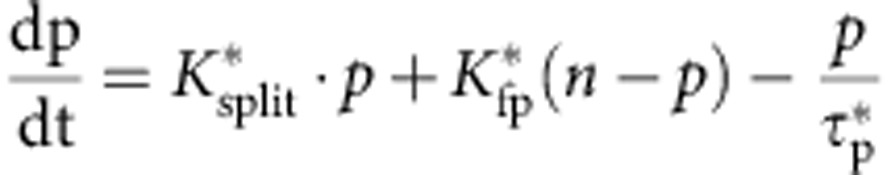



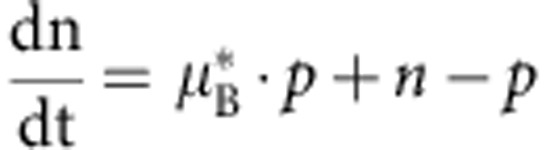


Where the asterisks indicate that the rate constants are normalized per division (or per generation). We first consider the case where 
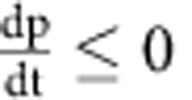
. In this case, *n* will increase exponentially and *p* will remain constant as 
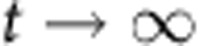
. Thus, the ratio 
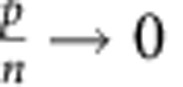
 as 
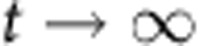
 in this case. The more interesting case is when 
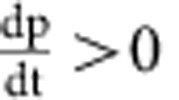
. It is fairly obvious that 
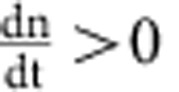
 because *n*>*p*. Thus, as 
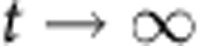
, both *p* and *n* will go to infinity. Since we are interested in the ratio *p*/*n*, we can use L'Hospital's rule:


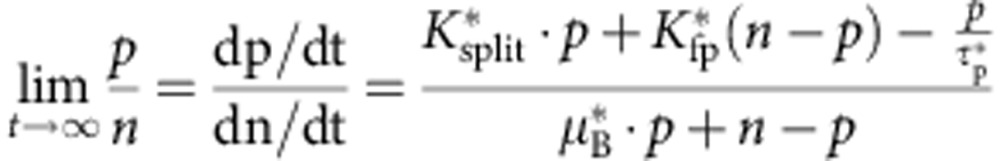


We know that at the steady state 
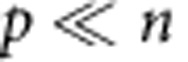
, usually by at least a factor of 100, thus, we can simplify by dividing everything by *n*:


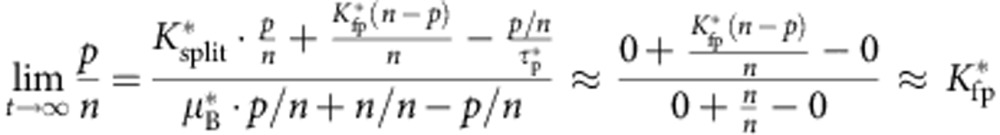


Thus, the dynamic range of DCDC is limited entirely by the false production rate.

(2) If we normalize dn/dt so that we are looking at relative population growth, we see that


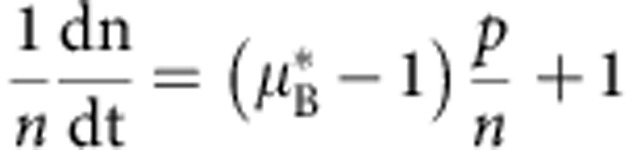


There is a trivial solution where 
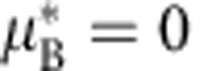
, and then nothing grows. Otherwise, this describes an exponential decay towards 1 (since eventually 
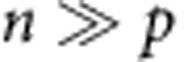
), which is the growth rate of dark cells. So, even if there is an initial difference between the growth rate of bright and dark cells, it will rapidly correct itself over time.

(3) This is similar to (2) but with the dp/dt equation:


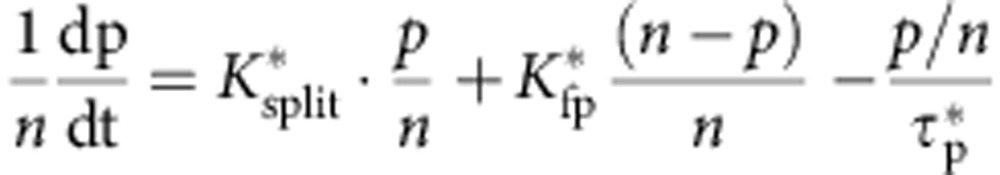


And we can see that splitting and degradation exponentially decrease in importance over time, whereas false production asymptotically increases towards its maximum value of *K*_fp_ for 
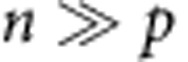
.

### Microbiota population dynamics modelling

We considered a total of seven cases in our model. Below, we show the equations used to describe the population dynamics in each of these cases:

- No feedback (Fig. 5a): 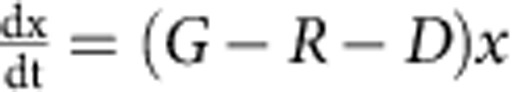

- Nutrient limitation (Fig. 5b): 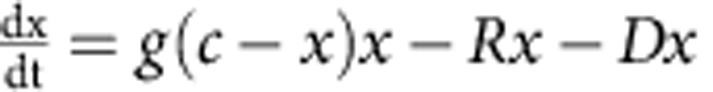

- Interactions with other bacteria (Fig. 5c): 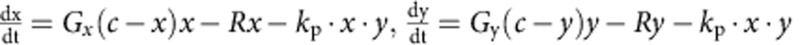

- Adaptation (Fig. 5d): 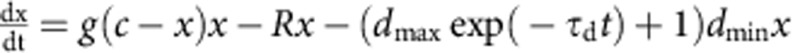

- Physiological removal (Supplementary Fig. 6a): 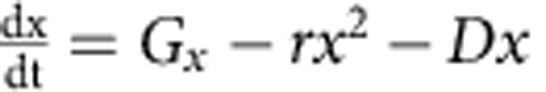

- Immune response (Supplementary Fig. 6b): 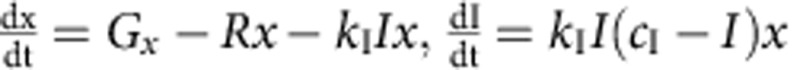

- Bacteriophage killing (Supplementary Fig. 6c): 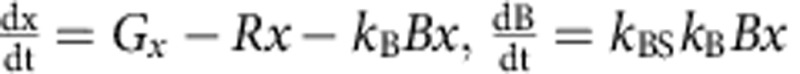

Here, *G* is the exponential growth rate, *R* is the removal rate, *D* is the death rate, *g* is the logistic growth rate, *c* is the carrying capacity, *G*_*x*_ and *G*_*y*_ are the logistic growth rates of species *x* and species *y*, respectively, *k*_p_ is the strength of the interaction between species *x* and *y*, (*d*_max_+1)*d*_min_ is the maximal death rate, *τ*_D_ is the rate at which the death rate decays towards the steady-state death rate, *d*_min_ is the steady-state death rate, *r* is the physiological removal rate, *k*_I_ is immune response rate, *c*_I_ is the maximal immune response level, *k*_B_ is the rate of bacteriophage infection, and *k*_BS_ is the burst size of the bacteriophage. We denote the bacterium of interest using *x*, the other species as *y*, the strength of the immune response using *I*, and the bacteriophages using *B*. We did not include a separate death term in the ‘interactions with other bacteria' case (Fig. 5c) as in this case death is mediated by interactions with other bacteria.

For the plots in Fig. 5a, we used *G=*1/3 h^−1^, *R=*1/6 h^−1^, and varied *D* from 0 to 1/3 h^−1^. For the plots in Fig. 5b, we used *g=*1/3 h^−1^, *R=*1/6 h^−1^, *c*=1 and varied *D* from 0 to 1/3 h^−1^. For the plots in Fig. 5c. we used *G*_*x*_=1/3 h^−1^, *G*_*y*_=½ h^−1^, *R=*1/6 h^−1^, and varied *k*_p_ from −1 to 1. For the plots in Fig. 5d, we used *g*=1/3 h^−1^, *R*=1/6 h^−1^, *d*_max_*=*30, *τ*_D=_0.5, and varied *d*_min_ from 0 to 1/3 h^−1^. For the plots in Supplementary Fig. 6a, we used *G*=1/3 h^−1^ *r*=1/3, and varied *D* from 0 to 1/3 h^−1^. For the plots in Supplementary Fig. 6b, we used *G*=1/3 h^−1^, *R*=1/6 h^−1^, and *c*_I_=1, and varied *k*_I_ from 0 to 1. For the plots in Supplementary Fig. 6c, we used *G*=1/3 h^−1^, *R*=1/6 h^−1^, *k*_BS_=50, and varied *k*_B_ from 0 to 1.

The simple cases (No feedback, nutrient limitation and physiological removal) were solved analytically using the DSolve function in Mathematica 10.2. All other cases were solved numerically using MATLAB.

## Additional information

**How to cite this article:** Myhrvold, C. *et al*. A distributed cell division counter reveals growth dynamics in the gut microbiota. *Nat. Commun.* 6:10039 doi: 10.1038/ncomms10039 (2015).

## Supplementary Material

Supplementary Information Supplementary Figures 1-7, Supplementary Tables 1-5 and Supplementary References

Supplementary Movie 1 DCDC particlces segregate faithfully in a bacterial microcolony. We have overlaid the brightfield (grayscale) and RFP channels (red) to show particle segregation over time. One frame is taken every five minutes as a colony of *E. coli* cells forms under an agar pad. The field of view is 37.38 μm on each side.

Supplementary Software 1 Turbidostat source code.

Supplementary Software 2 Flow cytometry data analysis scripts.
