# Supplementary material for: A distributed cell division counter reveals growth dynamics in the gut microbiota
Source: Nat Commun. 2015 Nov 30;6:10039. doi: 10.1038/ncomms10039 (PMC4674677; doi:10.1038/ncomms10039)
Supplement: Supplementary Information — Supplementary Figures 1-7, Supplementary Tables 1-5 and Supplementary References [file ncomms10039-s1.pdf]

Supplementary Information

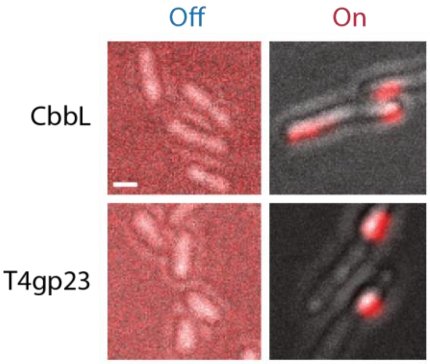

Supplementary Figure 1. Imaging additional SAP-RFP variants. Microscopy data for two additional variants (CbbL, and T4gp23) which produce heterogeneously sized particles are shown. The left column consists of uninduced cells, and the right column consists of cells which have been induced for 3 hours with 1 mM arabinose. Scale bar: 1 µm.

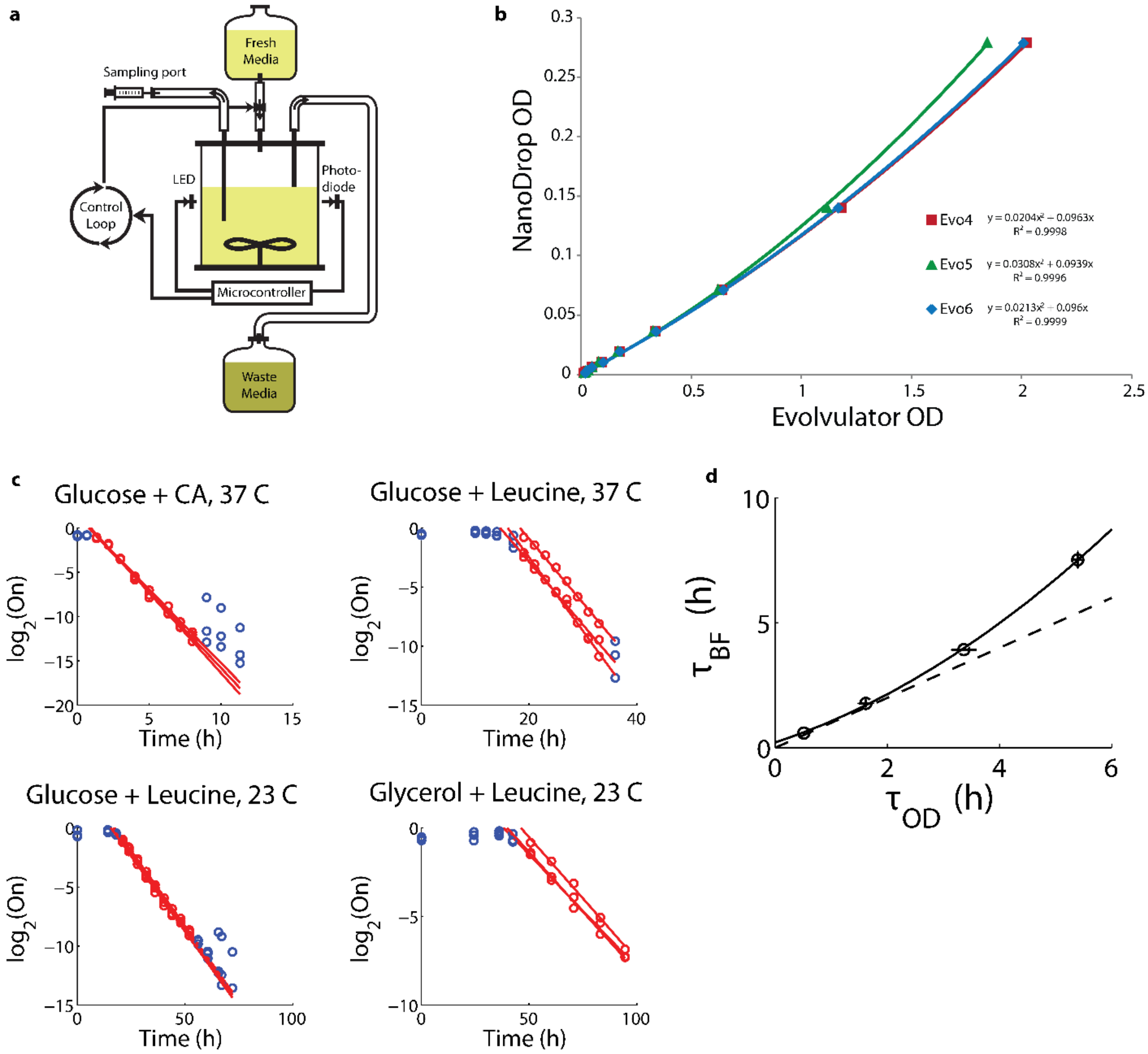

Supplementary Figure 2. Additional data from the turbidostat experiments. A detailed schematic of our home-built turbidostat is shown (a). We calibrated three turbidostats (Evo4, Evo5, Evo6) using serial dilutions of *E. coli* cells to ensure that readings were accurate across all machines (b). We also show the $\log_2$ of the fraction of cells in the "on" state over time from experiments under 4 different conditions, which we used to generate the plot in Fig. 2d (c). Red lines are linear fits to the red points in the dataset. Blue points are not used for curve fitting. We calculated the doubling time based on optical density (τ_{OD}) and the doubling time based on the curve fits from panel c (τ_{BF}) and compared them (black circles, d). Error bars indicate one standard deviation. The dashed line is x=y. The black line is a quadratic fit to the data, indicating that we can calibrate τ_{OD} and τ_{BF}.

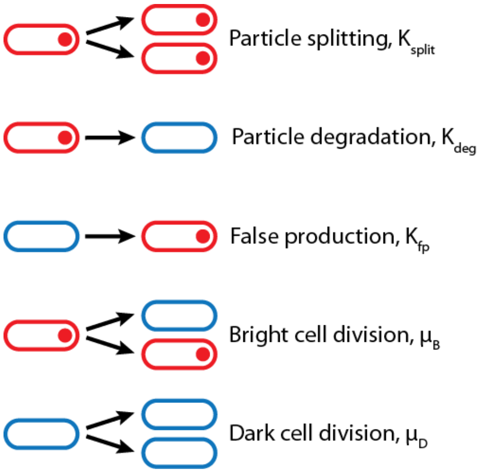

Supplementary Figure 3. Reactions in the error rate model and their corresponding rate coefficients. The diagrams indicate what is happening to the number of cells and particles, and are accompanied by a description of the phenomenon and the relevant parameter in the model.

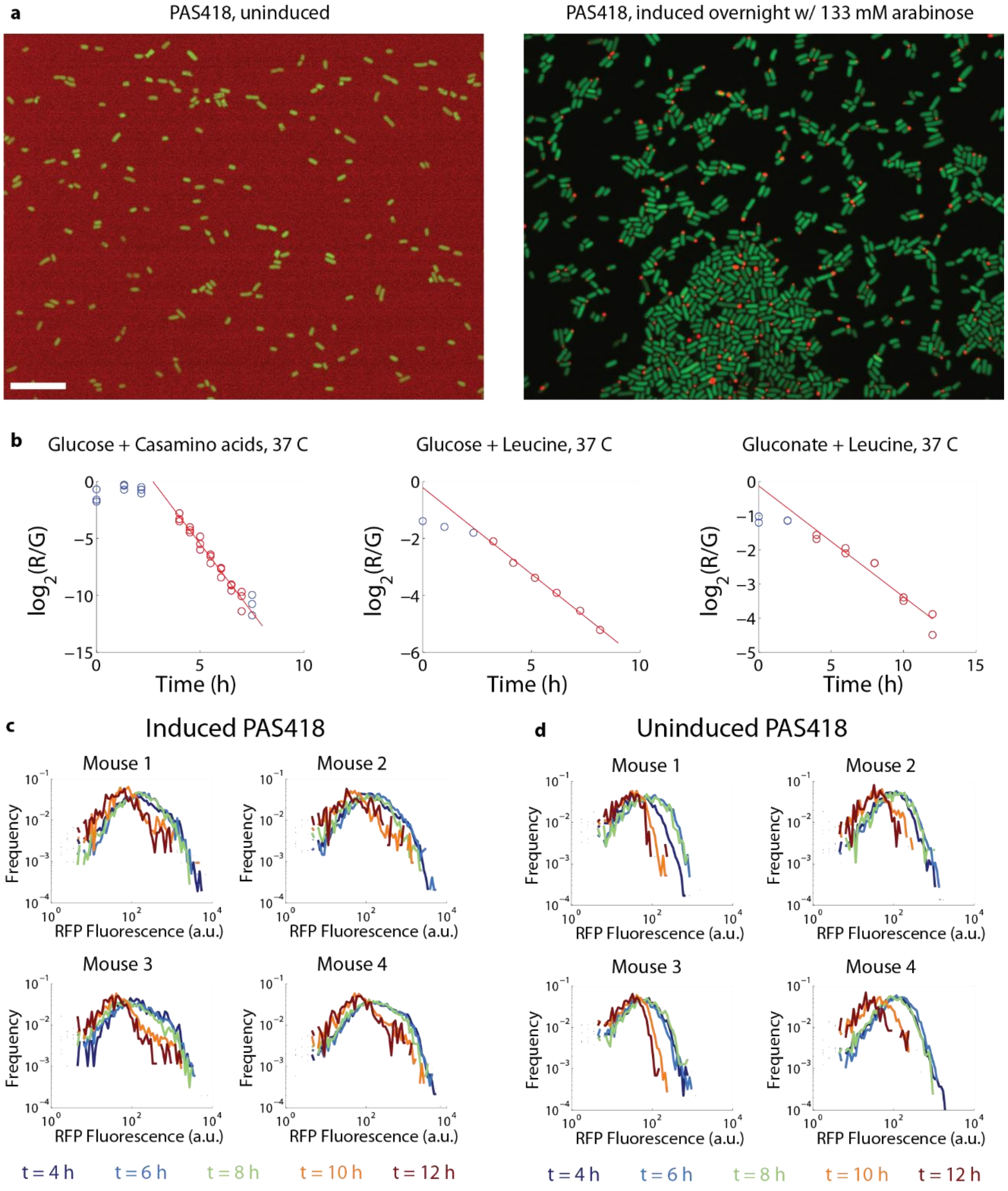

Supplementary Figure 4. Additional characterization of PAS418 *in vitro* and *in vivo*. We show large-scale images our engineered mouse-derived *E. coli* strain PAS418 with and without induction (a). Scale bar: 10 µm. We also show the $\log_2$ of the R/G fraction over time from turbidostat experiments under 3 different conditions, which was used to generate the plot in Fig. 3d (b). Raw flow cytometry data from the RFP channel from the experiments shown in Fig. 4g (induced PAS418 cells, c) and Fig. 4h (uninduced PAS418 cells, d) are shown. Each mouse is shown as a separate panel, with each time point shown in a different color. At most time points, the number of induced cells was less than 1 in 1,000. The data are plotted on a log-log scale.

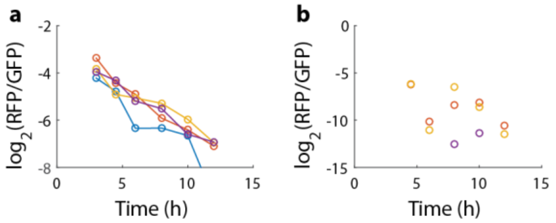

Supplementary Figure 5. Data from a replicate mouse experiment. We show data from a replicate experiment where we introduced induced (a) or uninduced (b) PAS418 bacteria into mice by oral gavage. The $\log_2$ of the RFP/GFP ratio is shown over time. Each color indicates a different mouse. In (a), data points are joined by lines to aid the eye.

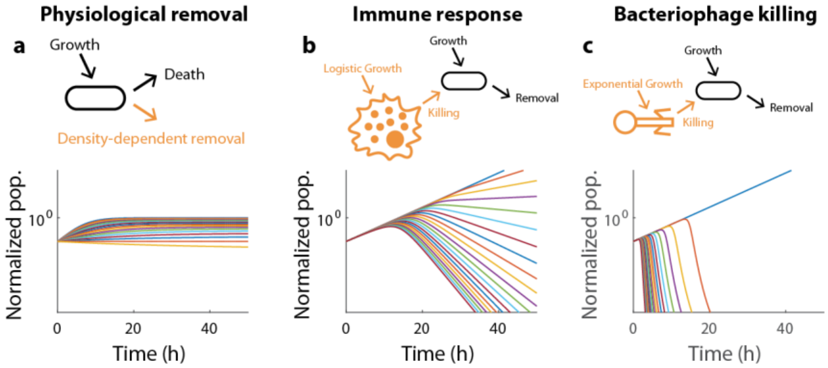

Supplementary Figure 6. Additional cases we analyzed in our microbiotal population dynamics model. We consider physiological feedback (density-dependent removal, a), an immune response (b), and bacteriophage-mediated killing (c). We systematically varied the removal rate in (a), the killing rate in (b), and the killing rate in (c).

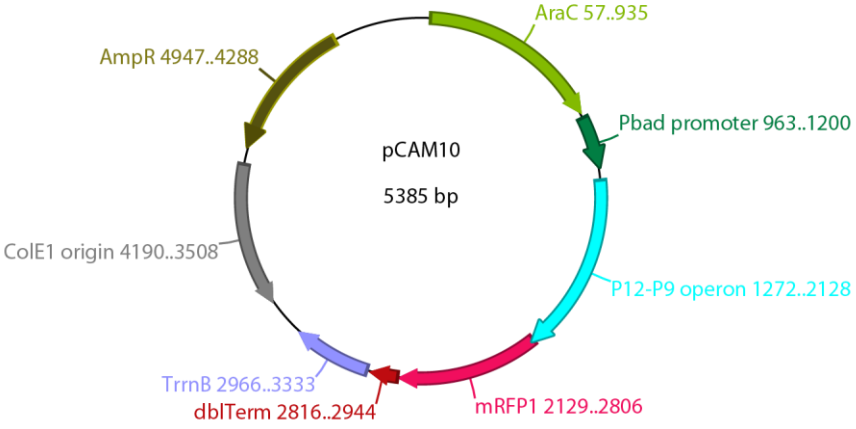

Supplementary Figure 7. Reference plasmid map. We show a plasmid map for pCAM10. Other plasmids used in this study are the same except for the self-assembling protein insert (cyan), which varies as discussed in Supplementary Table 2.

Supplementary Table 1. Comparison of methods for *in vivo* population dynamics measurement.

**Method**	**rRNA FISH**[a]	**WITS**[b]	**STAMP**[c]	**FD**[d]	**TIMER**[e]	**PTR**[f]	**Temperature-sensitive Plasmids**	**DCDC**[g]
Population / Single Cell	Population	Population	Population	Single cell	Single cell	Population	Population	Population
Genetic modification?	No	Yes	Yes	Yes	Yes	No	Yes	Yes
Directly measures growth?	No	No	No	Yes	Yes	No	Yes	Yes
Growth Rate Range	Not tested	Not tested	Not tested	Not tested	10-fold	Moderate	Not tested	10-fold
Multiplexing	Low	Medium	High	Low	Low	High	Low	Low
Other Limitations	Extensive calibration	Population size	Population size	Oxygen	Oxygen	Sequencing depth	*In vivo* temperature	Population size
References	1	2	3	4		5	6	This study

[a] rRNA fluorescent *in situ* hybridization
[b] Wild-type isogenic tagged strains
[c] Sequence tag-based analysis of microbial populations
[d] Fluorescence dilution
[e] TIMER is not an acronym, but is the name of a fluorescent protein
[f] Peak-to-trough ratio
[g] Distributed cell division counting.

Supplementary Table 2. Strains and plasmids used in this study.

**Strain Name (*Species*)**	**Genotype**	**Markers**	**Notes**	**Reference**
DP10 (*E. coli*)	F-, *mcr*A, Δ(*mrr-hsd*RMS-*mcr*BC), Φ80*lac*ZΔM15, Δ*lac*X74, *rec*A1, *end*A1, *ara*D139, Δ (*ara*, *leu*)7697, *gal*U, galK, λ-, rpsL (Str^R), *nup*G D(*ara*FGH) φ(D*ara*Ep P_{cp8}-*ara*E)	Str^R	Derived from DH10B. See reference for details.	[7]
PAS418 (*E. coli*)	*ara*B::Cm^R-tetP->cro, *mph*R::Kan^R-O_L-*rex*BA-cI^{ind-}-O_R-*cro*::*lac*Z, insO-1::pBF306->sfGFP pCAM10	Tet^R, Cm^R, Kan^R, $Strep^R$, Amp^R	Derived from PAS133[8]. Leucine auxotroph.	This study
Mach1 (*E. coli*)	F– Φ80*lac*ZΔM15 Δ*lac*X74 *hsd*R(rK–, mK+) Δ*rec*A1398 *end*A1 *ton*A	$T1^R$	Cloning strain, purchased from Invitrogen	N/A
**Plasmid Name**	**Expression construct**	**Markers**	**Notes**	**Reference**
pCAM10[h]	P_{ara}=>P12/P9-mRFP1	Amp^R	Expresses P12/P9-mRFP1	This study
pCAM44	P_{ara}=>*cbb*L-mRFP1	Amp^R	Expresses CbbL-mRFP1	This study
pCAM45	P_{ara}=>*cso*S1A-mRFP1	Amp^R	Expresses CsoS1A-mRFP1	This study
pCAM69	P_{ara}=>*pdu*A-mRFP1	Amp^R	Expresses PduA-mRFP1	This study
pCAM70	P_{ara}=>*eut*M-mRFP1	Amp^R	Expresses EutM-mRFP1	This study
pCAM72	P_{ara}=>T4_gp23-mRFP1	Amp^R	Expresses T4gp23-mRFP1	This study

[h] For a plasmid map of pCAM10, see Supplementary Figure 7.

Supplementary Table 3. Primer sequences used in this study.

Primer	Description	Sequence
**CP26**	pCAM10_pAra_p12p9_fwd	ACCCGCTAGCaaagaggagaaaAGAatggttatcggtctcctgaagtatctcac
**CP27**	pCAM10_pAra_p12p9_rev	gtgagatacttcaggagaccgataaccatTCTtttctcctctttGCTAGCGGGT
**CP28**	pCAM10_p12p9_mRFP1_fwd	gccaacatccctttcctggccatggcgagtagcgaagacgttatca
**CP29**	pCAM10_p12p9_mRFP1_rev	tgataacgtcttcgctactcgccatggccaggaaagggatgttggc
**CP129**	pCAM44_pAra_cbbL_fwd	CCCGCTAGCaaagaggagaaaAGAatggcagttaaaaagtatagtgctggtgt
**CP130**	pCAM44_pAra_cbbL_rev	acaccagcactatactttttaactgccatTCTtttctcctctttGCTAGCGGG
**CP131**	pCAM44_cbbL_mRFP1_fwd	tcgacaaactcgacactcaaaatcgtgcgagtagcgaagacgttatcaaaga
**CP132**	pCAM44_cbbL_mRFP1_rev	tctttgataacgtcttcgctactcgcacgattttgagtgtcgagtttgtcga
**CP133**	pCAM45_pAra_csoS1A_fwd	CCCGCTAGCaaagaggagaaaAGAatggctgatgtaactggtattgctct
**CP134**	pCAM45_pAra_csoS1A_rev	agagcaataccagttacatcagccatTCTtttctcctctttGCTAGCGGG
**CP135**	pCAM45_csoS1A_mRFP1_fwd	cctaaggcgccacaagccgcgagtagcgaagacgttatcaaaga
**CP136**	pCAM45_csoS1A_mRFP1_rev	tctttgataacgtcttcgctactcgcggcttgtggcgccttagg
**CP193**	pCAM69_pAra_pduA_fwd	TACCCGCTAGCaaagaggagaaaAGAatgcaacaagaagcactaggaatggt
**CP194**	pCAM69_pAra_pduA_rev	accattcctagtgcttcttgttgcatTCTtttctcctctttGCTAGCGGGTA
**CP195**	pCAM69_pduA_mRFP1_fwd	aaatcttaccgaagggaattagccaaatggcgagtagcgaagacgttat
**CP196**	pCAM69_pduA_mRFP1_rev	ataacgtcttcgctactcgccatttggctaattcccttcggtaagattt
**CP197**	pCAM70_pAra_eutM_fwd	TACCCGCTAGCaaagaggagaaaAGAatggaagcattaggaatgattgaaacc
**CP198**	pCAM70_pAra_eutM_rev	ggtttcaatcattcctaatgcttccatTCTtttctcctctttGCTAGCGGGTA
**CP199**	pCAM70_eutM_mRFP1_fwd	gcttcaaaggcgacagcaacattatggcgagtagcgaagacgttat
**CP200**	pCAM70_eutM_mRFP1_rev	ataacgtcttcgctactcgccataatgttgctgtcgcctttgaagc
**CP205**	pCAM72_pAra_T4gp23_fwd	TACCCGCTAGCaaagaggagaaaAGAatgactatcaaaactaaagctgaacttttgaaca
**CP206**	pCAM72_pAra_T4gp23_rev	tgttcaaaagttcagctttagttttgatagtcatTCTtttctcctctttGCTAGCGGGTA
**CP207**	pCAM72_T4gp23_mRFP1_fwd	cgcttactttagacgtgtatatgttaaaggtatcatggcgagtagcgaagacgttat
**CP208**	pCAM72_T4gp23_mRFP1_rev	ataacgtcttcgctactcgccatgatacctttaacatatacacgtctaaagtaagcg

Supplementary Table 4. Parts list to assemble one Evolvulator base station.

Item	Description	Manufacturer Part #	Supplier Catalog #	$ each	Qty.	Supplier
Arduino Clone	EtherTen	EtherTen		69.65	1	Freetronics
Diode	Diode 400V 1A	S1G	S1GFSCT-ND	0.53	6	Digi-Key
POT0	Potentiometers Flat 15mm 10k	RK09L1120036	688-RK09L1120036	0.98	1	Mouser
POT1	TRIMMER 100 OHM 0.5W PC PIN	3362M-1-101RLF	3362M-1-101RLF-ND	0.73	1	Digi-Key
Mosfet	MOSFET N-CH 30V 1.7A SC70-3	AO7400	785-1084-1-ND	0.57	5	Digi-Key
J0	CONN JACK POWER 2.1MM PCB	PJ-102A	CP-102A-ND	0.78	1	Digi-Key
JX	36pin 0.1 in .5 tall breakaway header	69152-436HLF	649-69152-436HLF	1.26	28 total pins	Mouser
JX	36pin 0.1 in .23 tall breakaway header	68000-236HLF	649-68000-236HLF	0.57	30 total pins	Mouser
Resistor0	1 kOhm 0805 1%	ERJ-6ENF1001V	P1.00KCCT-ND	0.07	2	Digi-Key
Resistor1	0 Ohm 0805	CRCW080500 00Z0EB	71-CRCW08050000Z0EB	0.07	1	Mouser
Resistor2	2.2 MOhm 0805 1%	RC0805FR-072M2L	311-2.20MCRCT-ND	0.02	1	Digi-Key
Capacitor	100 nF 0603	GRM188F51H104ZA01D	490-1568-1-ND	0.1	4	Digi-Key
Cables0	8" 3 Pin FLEX CABLE - AFF03G/AF03/AFF03G	A9BBG-0308F	A9BBG-0308F-ND	4.12	1	Digi-Key
Cables1	4" 3 Pin FLEX CABLE - AFF03G/AF03/AFF03G		A9BBG-0304F-ND	3.63	1	Digi-Key
Crimp receptacle	Molex KK 2695 3-position receptacle	22-01-2031	WM1576-ND	0.38	2	Digi-Key
wire crimps	CONN TERM FEMALE 22-30AWG TIN	08-50-0114	WM1114-ND	0.31	4	Digi-Key
LED0	green led 527 nm, 53650mcd, 15 deg 16800 mcd min, 34000 mcd typical	C503B-GAN-CB0F0791	C503B-GAN-CB0F0791-ND	0.23	1	Digi-Key
Sensor0	PhotoDiode with Amp	OPT101P	595-	6.88	1	Mouser

			OPT101P			
Valve0	Pinch Valve 12V 300 mA	100P2NC12-05S		86.05	1	Reet Corp.
Valve Bracket	Universal flange for 1 inch diameter valves or pumps		MU-100	5.36	1	Reet Corp.
PSU	12 V POWER supply wall wart 2.0A	EPSA120200U-P5P-SZ	T1071-P5P-ND	14.7	1	Digi-Key
FAN	12 V FAN 0.230A 4600 RPM 25.0 CFM (0.708m³/min) 60mm x 60mm x 25mm	OD6025-12HSS	1053-1238-ND	4.74	1	Digi-Key
Standoff1	Aluminum Threaded standoff FF 4-40 1.5in		91780A171	0.85	6	McMaster Carr
Standoff2	Aluminum Threaded standoff FF 4-40 2in		91780A174	0.97	8	McMaster Carr
bolt0	18-8 SS M4 x 30mm bolt 100 pack		91292A130	7.56	2	McMaster Carr
bolt1	18-8 SS M3 x 10mm bolt 100 pack		91292A113	5.85	2	McMaster Carr
bolt2	18-8 SS M3 x 8mm bolt 50 pack		92290A113	9.63	8	McMaster Carr
screw0	18-8 SS 4-40 BHCS L = 3/8" 100 pk		92949A108	1.47	8	McMaster Carr
screw1	18-8 SS 4-40 BHCS L = 0.5" 100 pk		92949A110	4.13	4	McMaster Carr
screw2	18-8 SS 4-40 BHCS L = 3/4" 100 pk		92949A113	4.55	2	McMaster Carr
nut0	18-8 SS M3 nut 100 pack		91828A211	5.55	13	McMaster Carr
nut1	18-8 SS M4 nut 100 pack		91828A231	6.45	14	McMaster Carr
panel1	Fluorescent black cast acrylic sheet 0.118" thick 24"x36"		8505K153	31.5	1	McMaster Carr
magnet0	1 1/2" x 1/2" x 1/8" thick neodymium magnet		BX882-N52	4.72	1	KJ Magnetics

Supplementary Table 5. Parts list for one bioreactor setup.

Item	Description	Manufacturer Part #	Supplier Catalog #	$ each	Qty.	Supplier
Cap	Screw cap GL45 3-port 2/pk	11 297 51	NC9690398	65.69	1	Fisher Scientific
Cap	Blank sealing screw cap 2/pk	11 562 92	NC9069754	4.85	1	Fisher Scientific
Cap	GL14 Screw cap for hose connection 2/pk	11 298 14	NC9785858	13.71	2	Fisher Scientific
compression fitting	GL14 Screw cap insert for 6mm OD Tubing	11 298 18	NC9570377	14.73	2	Fisher Scientific
Tubing	304 SS Smooth-Bore Seamless Tubing 1/8" OD, .085" ID, .02" Wall, 1' Length		89895K212	9.21	4.75" /bioreactor	McMaster Carr
Tubing	High-Temperature Silicone Rubber Tubing Soft, 1/8" ID, 1/4" OD, 1/16" Wall, White		51135K16	0.67/ft		McMaster Carr
Tubing	High-Temperature Silicone Rubber Tubing Soft, 1/4" ID, 3/8" OD, 1/16" Wall, White		51135K28	0.95/ft		McMaster Carr
Media reservoir	20 L 5.5 gal. Polypropylene Carboys w/ Handles & Spigot		2319-0050	149.24	1	Spectrum
Cap	Filling/Venting Closure with 1/4 inch Fittings, PP	2162-0831		40.26	1	Spectrum
waste reservoir	20 L 5.5 gal. Polypropylene Carboys w/ Handles	2250-0050		99.67	1	Spectrum
Stir bar	Magnetic stir bar 2-1/2" X 1/2"	58948-986		15.12	1	VWR

Supplementary References

1. Rang, C. U. *et al.* Estimation of growth rates of Escherichia coli BJ4 in streptomycin-treated and previously germfree mice by in situ rRNA hybridization. *Clin. Diagn. Lab. Immunol.* **6,** 434–6 (1999).

2. Grant, A. J. *et al.* Modelling within-host spatiotemporal dynamics of invasive bacterial disease. *PLoS Biol.* **6,** 757–770 (2008).

3. Abel, S. *et al.* Sequence tag–based analysis of microbial population dynamics. *Nat. Methods* **12,** (2015).

4. Helaine, S. *et al.* Dynamics of intracellular bacterial replication at the single cell level. *Proc. Natl. Acad. Sci. U. S. A.* **107,** 3746–3751 (2010).

5. Korem, T. *et al.* Growth dynamics of gut microbiota in health and disease inferred from single metagenomic samples. *Science (80-.).* **349,** 1101–1106 (2015).

6. Benjamin, W. H., Hall, P., Roberts, S. J. & Briles, D. E. The primary effect of the Ity locus is on the rate of growth of Salmonella typhimurium that are relatively protected from killing. *J. Immunol.* **144,** 3143–3151 (1990).

7. Pitera, D. J., Paddon, C. J., Newman, J. D. & Keasling, J. D. Balancing a heterologous mevalonate pathway for improved isoprenoid production in Escherichia coli. *Metab. Eng.* **9,** 193–207 (2007).

8. Kotula, J. W. *et al.* Programmable bacteria detect and record an environmental signal in the mammalian gut. *Proc. Natl. Acad. Sci. U. S. A.* **111,** 4838–43 (2014).
