# Supplementary material for: A distributed cell division counter reveals growth dynamics in the gut microbiota
Source: Nat Commun. 2015 Nov 30;6:10039. doi: 10.1038/ncomms10039 (PMC4674677; doi:10.1038/ncomms10039)
Supplement: Supplementary Software 1 — Turbidostat source code. [file ncomms10039-s3.zip › Newest_Code_For_Evo_GitHub_Repo/Evolvulator/code/autognarls/service/flaskapp/static/flot/examples/ajax.html]

Flot Examples


# Flot Examples

Example of loading data dynamically with AJAX. Percentage change in GDP (source: Eurostat). Click the buttons below.

The data is fetched over HTTP, in this case directly from text
files. Usually the URL would point to some web server handler
(e.g. a PHP page or Java/.NET/Python/Ruby on Rails handler) that
extracts it from a database and serializes it to JSON.

-
data -

-
data -

-
data -

If you combine AJAX with setTimeout, you can poll the server
for new data.
