# Supplementary material for: A distributed cell division counter reveals growth dynamics in the gut microbiota
Source: Nat Commun. 2015 Nov 30;6:10039. doi: 10.1038/ncomms10039 (PMC4674677; doi:10.1038/ncomms10039)
Supplement: Supplementary Software 1 — Turbidostat source code. [file ncomms10039-s3.zip › Newest_Code_For_Evo_GitHub_Repo/Evolvulator/code/autognarls/service/flaskapp/static/flot/examples/annotating.html]

Flot Examples


# Flot Examples

Flot has support for simple background decorations such as
lines and rectangles. They can be useful for marking up certain
areas. You can easily add any HTML you need with standard DOM
manipulation, e.g. for labels. For drawing custom shapes there is
also direct access to the canvas.
