# Supplementary material for: A distributed cell division counter reveals growth dynamics in the gut microbiota
Source: Nat Commun. 2015 Nov 30;6:10039. doi: 10.1038/ncomms10039 (PMC4674677; doi:10.1038/ncomms10039)
Supplement: Supplementary Software 1 — Turbidostat source code. [file ncomms10039-s3.zip › Newest_Code_For_Evo_GitHub_Repo/Evolvulator/code/autognarls/service/flaskapp/static/flot/examples/basic.html]

Flot Examples


# Flot Examples

Simple example. You don't need to specify much to get an
attractive look. Put in a placeholder, make sure you set its
dimensions (otherwise the plot library will barf) and call the
plot function with the data. The axes are automatically
scaled.
