# Supplementary material for: A distributed cell division counter reveals growth dynamics in the gut microbiota
Source: Nat Commun. 2015 Nov 30;6:10039. doi: 10.1038/ncomms10039 (PMC4674677; doi:10.1038/ncomms10039)
Supplement: Supplementary Software 1 — Turbidostat source code. [file ncomms10039-s3.zip › Newest_Code_For_Evo_GitHub_Repo/Evolvulator/code/autognarls/service/flaskapp/static/flot/examples/image.html]

Flot Examples


# Flot Examples

The Cat's Eye Nebula (picture from Hubble).

With the image plugin, you can plot images. This is for example
useful for getting ticks on complex prerendered visualizations.
Instead of inputting data points, you put in the images and where
their two opposite corners are supposed to be in plot space.

Images represent a little further complication because you need
to make sure they are loaded before you can use them (Flot skips
incomplete images). The plugin comes with a couple of helpers
for doing that.
