# Supplementary material for: A distributed cell division counter reveals growth dynamics in the gut microbiota
Source: Nat Commun. 2015 Nov 30;6:10039. doi: 10.1038/ncomms10039 (PMC4674677; doi:10.1038/ncomms10039)
Supplement: Supplementary Software 1 — Turbidostat source code. [file ncomms10039-s3.zip › Newest_Code_For_Evo_GitHub_Repo/Evolvulator/code/autognarls/service/flaskapp/static/flot/examples/index.html]

Flot Examples


# Flot Examples

Here are some examples for Flot, the Javascript charting library for jQuery:

- Basic example
- Different graph types
- Setting various options and annotating a chart
- Updating graphs with AJAX and real-time updates

Being interactive:

- Turning series on/off
- Rectangular selection support and zooming and zooming with overview (both with selection plugin)
- Interacting with the data points
- Panning and zooming (with navigation plugin)
- Automatically redraw when window is resized (with resize plugin)

Various features:

- Using other symbols than circles for points (with symbol plugin)
- Plotting time series and visitors per day with zooming and weekends (with selection plugin)
- Multiple axes and interacting with the axes
- Thresholding the data (with threshold plugin)
- Stacked charts (with stacking plugin)
- Using filled areas to plot percentiles (with fillbetween plugin)
- Tracking curves with crosshair (with crosshair plugin)
- Plotting prerendered images (with image plugin)
- Pie charts (with pie plugin)
