# Supplementary material for: A distributed cell division counter reveals growth dynamics in the gut microbiota
Source: Nat Commun. 2015 Nov 30;6:10039. doi: 10.1038/ncomms10039 (PMC4674677; doi:10.1038/ncomms10039)
Supplement: Supplementary Software 1 — Turbidostat source code. [file ncomms10039-s3.zip › Newest_Code_For_Evo_GitHub_Repo/Evolvulator/code/autognarls/service/flaskapp/static/flot/examples/interacting.html]

Flot Examples


# Flot Examples

One of the goals of Flot is to support user interactions. Try
pointing and clicking on the points.

Mouse hovers at
(0, 0).

A tooltip is easy to build with a bit of jQuery code and the
data returned from the plot.

Enable tooltip
