# Supplementary material for: A distributed cell division counter reveals growth dynamics in the gut microbiota
Source: Nat Commun. 2015 Nov 30;6:10039. doi: 10.1038/ncomms10039 (PMC4674677; doi:10.1038/ncomms10039)
Supplement: Supplementary Software 1 — Turbidostat source code. [file ncomms10039-s3.zip › Newest_Code_For_Evo_GitHub_Repo/Evolvulator/code/autognarls/service/flaskapp/static/flot/examples/multiple-axes.html]

Flot Examples


# Flot Examples

Multiple axis support showing the raw oil price in US $/barrel of
crude oil vs. the exchange rate from US $ to €.

As illustrated, you can put in multiple axes if you
need to. For each data series, simply specify the axis number.
In the options, you can then configure where you want the extra
axes to appear.

Position axis left or right.
