# Supplementary material for: A distributed cell division counter reveals growth dynamics in the gut microbiota
Source: Nat Commun. 2015 Nov 30;6:10039. doi: 10.1038/ncomms10039 (PMC4674677; doi:10.1038/ncomms10039)
Supplement: Supplementary Software 1 — Turbidostat source code. [file ncomms10039-s3.zip › Newest_Code_For_Evo_GitHub_Repo/Evolvulator/code/autognarls/service/flaskapp/static/flot/examples/navigate.html]

Flot Examples


# Flot Examples

With the navigate plugin it is easy to add panning and zooming.
Drag to pan, double click to zoom (or use the mouse scrollwheel).

The plugin fires events (useful for synchronizing several
plots) and adds a couple of public methods so you can easily build
a little user interface around it, like the little buttons at the
top right in the plot.
