# Supplementary material for: A distributed cell division counter reveals growth dynamics in the gut microbiota
Source: Nat Commun. 2015 Nov 30;6:10039. doi: 10.1038/ncomms10039 (PMC4674677; doi:10.1038/ncomms10039)
Supplement: Supplementary Software 1 — Turbidostat source code. [file ncomms10039-s3.zip › Newest_Code_For_Evo_GitHub_Repo/Evolvulator/code/autognarls/service/flaskapp/static/flot/examples/percentiles.html]

Flot Examples


# Flot Examples

Height in centimeters of individuals from the US (2003-2006) as function of
age in years (source: CDC).
The 15%-85%, 25%-75% and 50% percentiles are indicated.

For each point of a filled curve, you can specify an arbitrary
bottom. As this example illustrates, this can be useful for
plotting percentiles. If you have the data sets available without
appropriate fill bottoms, you can use the fillbetween plugin to
compute the data point bottoms automatically.
