# Supplementary material for: A distributed cell division counter reveals growth dynamics in the gut microbiota
Source: Nat Commun. 2015 Nov 30;6:10039. doi: 10.1038/ncomms10039 (PMC4674677; doi:10.1038/ncomms10039)
Supplement: Supplementary Software 1 — Turbidostat source code. [file ncomms10039-s3.zip › Newest_Code_For_Evo_GitHub_Repo/Evolvulator/code/autognarls/service/flaskapp/static/flot/examples/resize.html]

Flot Examples


# Flot Examples

Sometimes it makes more sense to just let the plot take up the
available space. In that case, we need to redraw the plot each
time the placeholder changes its size. If you include the resize
plugin, this is handled automatically.

Try resizing the window.
