# Supplementary material for: A distributed cell division counter reveals growth dynamics in the gut microbiota
Source: Nat Commun. 2015 Nov 30;6:10039. doi: 10.1038/ncomms10039 (PMC4674677; doi:10.1038/ncomms10039)
Supplement: Supplementary Software 1 — Turbidostat source code. [file ncomms10039-s3.zip › Newest_Code_For_Evo_GitHub_Repo/Evolvulator/code/autognarls/service/flaskapp/static/flot/examples/selection.html]

Flot Examples


# Flot Examples

1000 kg. CO2 emissions per year per capita for various countries (source: Wikipedia).

Flot supports selections through the selection plugin.
You can enable rectangular selection
or one-dimensional selection if the user should only be able to
select on one axis. Try left-click and drag on the plot above
where selection on the x axis is enabled.

You selected:

The plot command returns a plot object you can use to control
the selection. Click the buttons below.

Selections are really useful for zooming. Just replot the
chart with min and max values for the axes set to the values
in the "plotselected" event triggered. Enable the checkbox
below and select a region again.

Zoom to selection.
