# Supplementary material for: A distributed cell division counter reveals growth dynamics in the gut microbiota
Source: Nat Commun. 2015 Nov 30;6:10039. doi: 10.1038/ncomms10039 (PMC4674677; doi:10.1038/ncomms10039)
Supplement: Supplementary Software 1 — Turbidostat source code. [file ncomms10039-s3.zip › Newest_Code_For_Evo_GitHub_Repo/Evolvulator/code/autognarls/service/flaskapp/static/flot/examples/setting-options.html]

Flot Examples


# Flot Examples

There are plenty of options you can set to control the precise
looks of your plot. You can control the ticks on the axes, the
legend, the graph type, etc. The idea is that Flot goes to great
lengths to provide sensible defaults so that you don't have to
customize much for a good result.
