# Supplementary material for: A distributed cell division counter reveals growth dynamics in the gut microbiota
Source: Nat Commun. 2015 Nov 30;6:10039. doi: 10.1038/ncomms10039 (PMC4674677; doi:10.1038/ncomms10039)
Supplement: Supplementary Software 1 — Turbidostat source code. [file ncomms10039-s3.zip › Newest_Code_For_Evo_GitHub_Repo/Evolvulator/code/autognarls/service/flaskapp/static/flot/examples/thresholding.html]

Flot Examples


# Flot Examples

With the threshold plugin, you can apply a specific color to
the part of a data series below a threshold. This is can be useful
for highlighting negative values, e.g. when displaying net results
or what's in stock.
