# Supplementary material for: A distributed cell division counter reveals growth dynamics in the gut microbiota
Source: Nat Commun. 2015 Nov 30;6:10039. doi: 10.1038/ncomms10039 (PMC4674677; doi:10.1038/ncomms10039)
Supplement: Supplementary Software 1 — Turbidostat source code. [file ncomms10039-s3.zip › Newest_Code_For_Evo_GitHub_Repo/Evolvulator/code/autognarls/service/flaskapp/static/flot/examples/time.html]

Flot Examples


# Flot Examples

Monthly mean atmospheric CO2 in PPM at Mauna Loa, Hawaii (source: NOAA/ESRL).

If you tell Flot that an axis represents time, the data will
be interpreted as timestamps and the ticks adjusted and
formatted accordingly.

Zoom to: Whole period
1990-2000
1999

The timestamps must be specified as Javascript timestamps, as
milliseconds since January 1, 1970 00:00. This is like Unix
timestamps, but in milliseconds instead of seconds (remember to
multiply with 1000!).

As an extra caveat, the timestamps are interpreted according to
UTC to avoid having the graph shift with each visitor's local
time zone. So you might have to add your local time zone offset
to the timestamps or simply pretend that the data was produced
in UTC instead of your local time zone.
