# Supplementary material for: A distributed cell division counter reveals growth dynamics in the gut microbiota
Source: Nat Commun. 2015 Nov 30;6:10039. doi: 10.1038/ncomms10039 (PMC4674677; doi:10.1038/ncomms10039)
Supplement: Supplementary Software 1 — Turbidostat source code. [file ncomms10039-s3.zip › Newest_Code_For_Evo_GitHub_Repo/Evolvulator/code/autognarls/service/flaskapp/static/flot/examples/tracking.html]

Flot Examples


# Flot Examples

You can add crosshairs that'll track the mouse position, either
on both axes or as here on only one.

If you combine it with listening on hover events, you can use
it to track the intersection on the curves by interpolating
the data points (look at the legend).
