# Supplementary material for: A distributed cell division counter reveals growth dynamics in the gut microbiota
Source: Nat Commun. 2015 Nov 30;6:10039. doi: 10.1038/ncomms10039 (PMC4674677; doi:10.1038/ncomms10039)
Supplement: Supplementary Software 1 — Turbidostat source code. [file ncomms10039-s3.zip › Newest_Code_For_Evo_GitHub_Repo/Evolvulator/code/autognarls/service/flaskapp/static/flot/examples/turning-series.html]

Flot Examples


# Flot Examples

Here is an example with real data: military budgets for
various countries in constant (2005) million US dollars (source: SIPRI).

Since all data is available client-side, it's pretty easy to
make the plot interactive. Try turning countries on/off with the
checkboxes below.

Show:
