# Supplementary material for: A distributed cell division counter reveals growth dynamics in the gut microbiota
Source: Nat Commun. 2015 Nov 30;6:10039. doi: 10.1038/ncomms10039 (PMC4674677; doi:10.1038/ncomms10039)
Supplement: Supplementary Software 1 — Turbidostat source code. [file ncomms10039-s3.zip › Newest_Code_For_Evo_GitHub_Repo/Evolvulator/code/autognarls/service/flaskapp/templates/error404.html]

{% extends "layout.html" %}
{% block title %}Woah Boy{% endblock %}
{% block body %}

## Evolvulator Whoops

# Page Not Found

{{ message }}

Try from the start
{% endblock %}
