# Supplementary material for: A distributed cell division counter reveals growth dynamics in the gut microbiota
Source: Nat Commun. 2015 Nov 30;6:10039. doi: 10.1038/ncomms10039 (PMC4674677; doi:10.1038/ncomms10039)
Supplement: Supplementary Software 1 — Turbidostat source code. [file ncomms10039-s3.zip › Newest_Code_For_Evo_GitHub_Repo/Evolvulator/code/autognarls/service/flaskapp/templates/experiment.html]

{% extends "layout.html" %}
{% block title %}Experiment{% endblock %}
{% block body %}

|  |  |  |  |  |  |  |  |
| --- | --- | --- | --- | --- | --- | --- | --- |
| Evolvulator Parameters for {{core.jobname}} @ {{core.url}}  | Name | Value | Note | | --- | --- | --- | {% for item in parameters %}| {{ item.key }} |  | {{ item.note }} | {% else %}|*Unbelievable. No params here so far* {% endfor %} | Optical Density   Time Coordinates: (0, 0) |

### System Messages:

?

|  |  |  |
| --- | --- | --- |
| UPDATE | START | LOOP |

 
{% endblock %}
