# Supplementary figures and images for: A distributed cell division counter reveals growth dynamics in the gut microbiota
Source: Nat Commun. 2015 Nov 30;6:10039. doi: 10.1038/ncomms10039 (PMC4674677; doi:10.1038/ncomms10039)

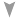

Supplement: Supplementary Software 1 — Turbidostat source code. [file ncomms10039-s3.zip › Newest_Code_For_Evo_GitHub_Repo/Evolvulator/code/autognarls/service/flaskapp/static/flot/examples/arrow-down.gif]

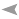

Supplement: Supplementary Software 1 — Turbidostat source code. [file ncomms10039-s3.zip › Newest_Code_For_Evo_GitHub_Repo/Evolvulator/code/autognarls/service/flaskapp/static/flot/examples/arrow-left.gif]

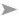

Supplement: Supplementary Software 1 — Turbidostat source code. [file ncomms10039-s3.zip › Newest_Code_For_Evo_GitHub_Repo/Evolvulator/code/autognarls/service/flaskapp/static/flot/examples/arrow-right.gif]

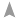

Supplement: Supplementary Software 1 — Turbidostat source code. [file ncomms10039-s3.zip › Newest_Code_For_Evo_GitHub_Repo/Evolvulator/code/autognarls/service/flaskapp/static/flot/examples/arrow-up.gif]

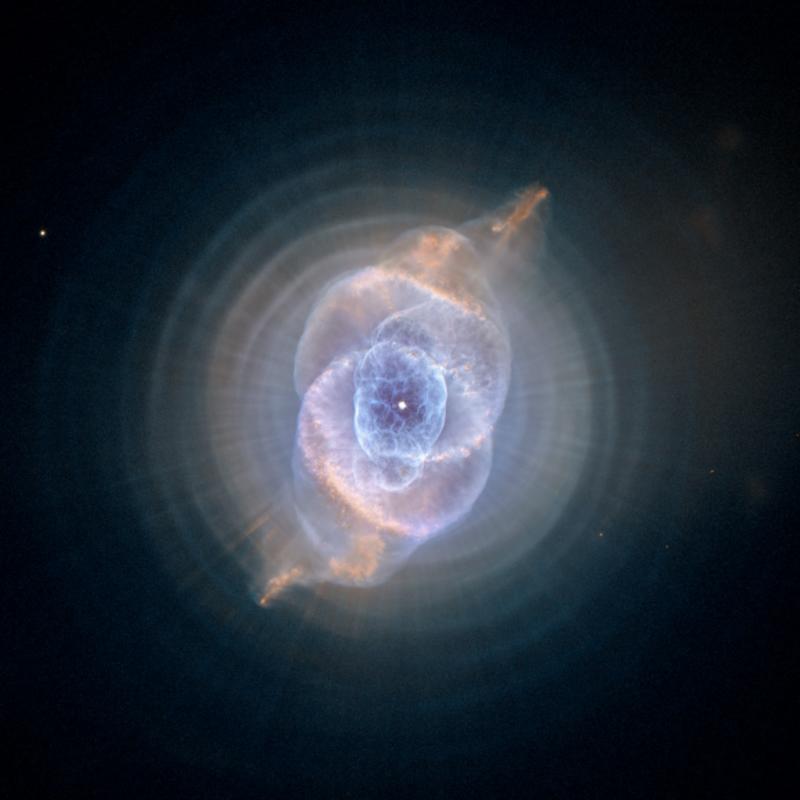

Supplement: Supplementary Software 1 — Turbidostat source code. [file ncomms10039-s3.zip › Newest_Code_For_Evo_GitHub_Repo/Evolvulator/code/autognarls/service/flaskapp/static/flot/examples/hs-2004-27-a-large_web.jpg]
