# Supplementary material for: A distributed cell division counter reveals growth dynamics in the gut microbiota
Source: Nat Commun. 2015 Nov 30;6:10039. doi: 10.1038/ncomms10039 (PMC4674677; doi:10.1038/ncomms10039)
Supplement: Supplementary file 105 [file ncomms10039-s3.zip › Newest_Code_For_Evo_GitHub_Repo/Evolvulator/Evolvulator documentation.odt]

Evolvulator software documentation

Server dependencies include: Python 2.7, Twisted, and Flask

Setting up the Evolvulator server:

Create a new database file:

- navigate to /Evolvulator/code/autocharles/service/experimentcore

- run python

- type “from exp_dbifc import createDB” without quotes

- type “createDB(“/location/of/created/database/file_name.DB”)” don't type outside-most quotes

  - replace /location/of/created/database/ with the path to the directory where the database will be created

  - replace file_name with the name of the database.

  - e.g. /home/wade/Documents/Evolvulator/database/Wade.DB

start the server with the following command:

twistd -noy app.tac 

to output information displayed in the terminal to a log file initiate the server with the following command:

twistd -noy app.tac | tee logfile.txt or twistd -noy app.tac > logfile.txt
