# Supplementary material for: A distributed cell division counter reveals growth dynamics in the gut microbiota
Source: Nat Commun. 2015 Nov 30;6:10039. doi: 10.1038/ncomms10039 (PMC4674677; doi:10.1038/ncomms10039)
Supplement: Supplementary file 105 [file ncomms10039-s3.zip › Newest_Code_For_Evo_GitHub_Repo/Evolvulator/Evolvulator startup documentation.odt]

Bioreactor setup:

Connect small quick release tube to the stop cock of the media reservoirs and attach with a zip tie.

Connect bioreactor feeder tube to fresh media reservoir.

Connect bioreactor waste tube to waste reservoir. 

Connect equalizer tubing between waste and fresh media reservoirs.

Tape equalizer tubing to the side of media reservoir.

Tighten all ports on the top of bioreactor.

Fill bioreactor by opening media reservoir stop cock.

Once full place feeder tube into the pinch valve.

Check that feeder tubing is completely in the valve and make sure all connector tops on the top of the bioreactor are tight!

To Start server:

cd to /Documents/Avi_Test/code/autognarls/service

start server with twistd -noy app.tac > date_log.txt

chrome opens localhost:8080

open new tabs for Evo4-6

After filling each bioreactor with clean media, turn on the stir bar, and change the max sensor reading (blank) on the control web page with the strip chart.  You can determine the max sensor reading by opening a web page of each Evolvulator IP and looking at Analog 2.  Refresh a couple times to get accurate reading.

On the control web page hit the “Start” button to begin recording data to database.  Hit “Loop” to start control loop.

After experiment is complete, copy and rename the Wade.DB files to save all data.
